# Remodeling of ferroptotic necroinflammation by dexamethasone in acute tubular necrosis

**DOI:** 10.1038/s41419-026-09099-w

**Published:** 2026-07-15

**Authors:** Natalie Bethe, Mirela Tmava, Alix Bruneau, Karolin Flade, Benjamin Böhme, Erik Klapproth, Selina Michel, Marlena Nastassja Schlecht, Siyu Xu, Ali El-Armouche, Frank Tacke, Stefan R. Bornstein, Wulf Tonnus

**Affiliations:** 1 Medical Clinic and Policlinic 3, University Hospital CGC at the Technische Universität Dresden, Dresden, Germany; 2 Department of Hepatology and Gastroenterology, Campus Charité Mitte and Campus Virchow-Klinikum, Charité-Universitätsmedizin Berlin, Berlin, Germany; 3 Institute of Pharmacology and Toxicology, Technische Universität Dresden, Dresden, Germany; 4 Diabetes and Nutritional Sciences, King’s College London, London, UK

**Keywords:** Cell death, Endocrine system and metabolic diseases, Acute kidney injury

## Abstract

Ferroptosis, a modality of regulated necrosis caused by iron-dependent lipid peroxidation, has emerged as a key contributor to the pathogenesis of acute tubular necrosis (ATN) – especially in the context of solid organ transplantation. While recent studies have identified hormones as extracellular signals that modulate cellular susceptibility to ferroptosis, their pathophysiological relevance in vivo remains incompletely defined. Here, we investigated the role of dexamethasone, a potent synthetic glucocorticoid previously reported to sensitize cells to ferroptosis, in the context of renal tubular injury. Unexpectedly, we observed a biphasic effect: early sensitization to ferroptosis induced by glutathione depletion was subsequently counteracted by a marked increase in ferroptosis resistance. Mechanistically, we found that dexamethasone reduced availability of ferrous iron via effects of the glucocorticoid receptor 1 (GR1), which also resulted in attenuated renal tubular injury. However, this cytoprotective effect did not translate into improved renal function, as dexamethasone exerted additional ferroptosis-independent effects on innate immune cell recruitment to injured kidneys. Collectively, our findings highlight the highly dynamic nature of the ferroptotic process in which modulatory signals can exert temporally opposing effects. Moreover, they underscore the complex interplay between regulated cell death and innate immune responses in shaping necroinflammation in disease trajectories.

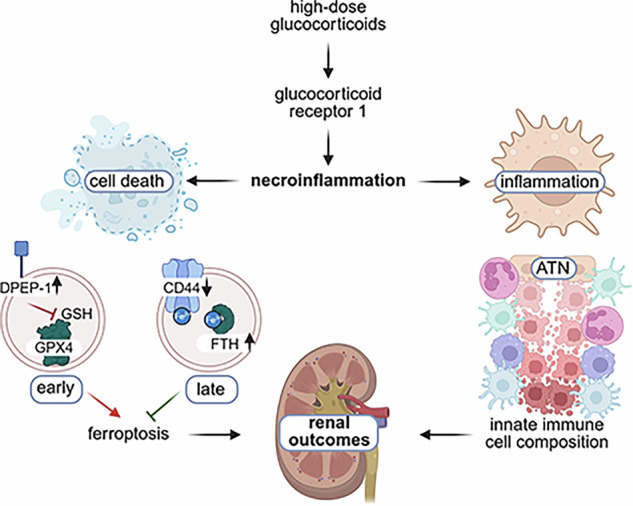

## Introduction

An estimated 850,000,000 people worldwide have chronic kidney disease [1], a condition associated with marked morbidity and mortality. End-stage kidney disease requires renal replacement therapy, which includes dialysis and, preferably, kidney transplantation. Inevitably, the surgical process of transplantation involves ischemia/reperfusion injury (IRI) causing trauma to the transplanted organ causing delayed graft function and chronic scarring impeding long-term organ function and graft survival [2]. Within the last years, it became evident that renal tubular ferroptosis, a modality of regulated necrosis caused by iron-dependent lipid peroxidation [3], is a pathophysiological hallmark of IRI, raising considerable interest in the development of preventive strategies upon solid organ transplantation [4]. Three interrelated factors largely define cellular ferroptosis susceptibility: Lipidome composition, availability of redox equivalents such as glutathione (GSH), and the labile iron pool. Intracellular ferrous iron (Fe^2+^) levels are tightly regulated through multiple mechanisms including import via transferrin receptor 1 and CD44, export via SLC40A1, storage via ferritin, lysosomal ferritinophagy mediated by NCOA4, and relocalization of iron to the cytosol [5].

Most mechanistic insights into ferroptosis were derived from cellular models and screens. While highly informative, these approaches frequently fail to capture the influence of systemic or extracellular signals such as estrogen [6] or glucocorticoids [7]. Importantly, high-dose glucocorticoids remain a mainstay of immunosuppressive induction therapy upon solid organ transplantation to prevent acute rejection [8], spurring interest to thoroughly characterize the effects of glucocorticoids on ferroptosis. We previously reported that the highly potent synthetic glucocorticoid dexamethasone promotes ferroptosis via glucocorticoid receptor 1 (GR1)-dependent upregulation of dipeptidase 1 (DPEP-1) resulting in reduced GSH availability. Whereas mechanistic work was performed in highly-mutated cancer cell lines, dexamethasone treatment translated to increased ferroptosis in isolated murine renal tubules [7]. However, how dexamethasone modulates ferroptosis in the complex, multicellular context of renal IRI remains unclear.

In situ, renal tubular ferroptosis typically manifests as acute tubular necrosis (ATN) [9, 10]. Furthermore, we previously demonstrated that ATN formation in vivo is strongly influenced by the recruitment and activation of innate immune cells [2, 11]. This bidirectional interplay between regulated cell death and inflammation has been conceptualized as necroinflammation [12]. Given that glucocorticoids are widely used immunosuppressive agents, it appears likely that they influence both sides of necroinflammation upon AKI [2, 12, 13].

In this study, we demonstrate that dexamethasone exerts temporally divergent effects on ferroptosis during AKI. While early ferroptosis sensitization is observed, dexamethasone unexpectedly reduces ferroptosis at later time points by limiting iron availability. This results in attenuated renal tubular necrosis in vivo but does not lead to improved renal function, as dexamethasone concurrently alters innate immune cell recruitment to injured kidneys. Together, our findings highlight the dynamic nature of ferroptosis and illustrate how therapeutic interference with necroinflammation can shape disease trajectories.

## Methods

### Cell lines and cell culture

The human proximal tubular cell line CD10-135 (referred to as CD10 in the manuscript) [14], originally provided by Prof. R. Kramann (RWTH Aachen), was cultured in Dulbecco’s Modified Eagle Medium F-12 Nutrient Mixture with GlutaMAX (DMEM/F12 GlutaMAX, Thermo Fisher, 10565018) supplemented with 10% (v/v) FBS (Thermo Fisher, 41966029), 100 U/ml penicillin, and 100 μg/ml streptomycin (Pen/Strep, Thermo Fisher, 15140122). The cells were incubated under humidified conditions at 37 °C with 5% CO₂.

### Plating and treatment of cells

CD10-135 cells were detached using Trypsin-EDTA (Gibco, 25200056), washed, and subsequently seeded into six-well plates (Sarstedt, 83.3920) at a density of 1 × 10⁵ cells per well. Before treatment, the culture medium was replaced. All experiments were conducted in a final volume of 1 ml.

### siRNA-mediated GR1 knockdown

CD10-135 cells were seeded on day 1 at a density of 9 × 10^5^ cells per 10 cm dish in DMEM/F12 GlutaMAX medium supplemented with 10% FBS without penicillin/streptomycin. On day 2, cells were transfected with either scrambled control siRNA (Silencer® Select Negative Control #1 siRNA, 4390843, Thermo Fisher Scientific) or siRNA targeting the glucocorticoid receptor (siGR) (s67066, Thermo Fisher Scientific) using Lipofectamine-mediated transfection. For each condition, 18 μl siRNA solution (180 pmol; diluted 1:10 in nuclease-free water) and 24 μl Lipofectamine^TM^ RNAiMAX (10514953, Thermo Fisher Scientific) were diluted separately in 1.5 ml Opti-MEM^TM^ Medium (10149832, Thermo Fisher Scientific). After incubation for 15 min at room temperature, both solutions were combined and added dropwise to the cells combined. On day 3, cells were washed with PBS, detached using trypsin, and replated into 6-well plates at a density of 1 × 10^5^ cells per well in complete DMEM/F12 GlutaMAX medium containing 10% FBS and penicillin/streptomycin. In parallel, additional cells were seeded into 10 cm dishes for subsequent protein isolation. In the evening of day 3, dexamethasone (1 µM, Hameln Pharma GmbH) pretreatment was initiated. On day 4, 48 h post-transfection, cells were treated with the ferroptosis-inducing compounds erastin, RSL3, or FINO2 in the presence or absence of Ferrostatin-1 (Fer-1) as indicated. Cells were harvested at defined time points depending on the respective treatment condition for flow cytometry analysis using annexin V and 7-AAD staining. In parallel, cell pellets were collected for Western blot analysis. GR1 knockdown efficiency was validated by immunoblotting using antibodies against glucocorticoid receptor 1 (12041, Cell Signaling) and beta-actin (3700 S, Cell Signaling) as loading control. All experiments were performed in three independent biological replicates. GR1-deficient HT1080 cells were published previously by us [7] and were used for control purposes.

### Cell death assays

The following ferroptosis inducers (FINs) were used: Type I FIN: erastin (Sigma Aldrich, #E7781) and sorafenib (biomol, #CAY1009644), type II FIN: RSL3 (Selleckchem, #S8155), type III FIN: FIN56 (Sigma Aldrich, #SML1740) and type IV FIN: FINO2 (Cayman Chemical, #25096). Fer-1 (Merck Millipore, #341494) and DFO (Selleckchem, #S5742) served as protection controls. Dexamethasone (Hameln Pharma GmbH) was pretreated for 16 h before adding FINs and inhibitors. Unless stated otherwise, the following concentrations were applied: 5 μM erastin, 1.13 μM RSL3, 10 μM FIN56, 10 μM FINO2, 10 μM sorafenib, 1 μM Fer-1, 50 μM DFO, and 1 μM dexamethasone. At the indicated time points, cells were harvested and processed for further workup.

### Flow cytometry

Cells were collected, washed twice with PBS, and stained using 5 μl of 7-AAD (BD Biosciences, #559925) and 5 μl of annexin V-FITC (BD Biosciences, #556420) in 100 μl of annexin V binding buffer (BD Biosciences, #556454) per sample. Following a 15-min incubation, samples were analyzed either on the Fortessa LSRII system using FACS Diva 6.1.1 software (BD Biosciences) or on the Symphony A3 system with FACS Diva v9.0 software (BD Biosciences). Data analysis was performed using FlowJo version 10. Flow cytometry was conducted with the support of the Flow Cytometry Core Facility of the CMCB Technology Platform and the FACS Facility of the Institute for Physiological Chemistry, both at the Technische Universität Dresden.

### BODIPY 581/591 C11 analysis

Lipid peroxidation was evaluated by staining harvested cells with 2 μM BODIPY 581/591 C11 (Thermo Fischer, #D3861) in 500 μl of Hanks’ Balanced Salt Solution (HBSS; Gibco/Life Technologies, #14175053) for 10 min at 37 °C. After staining, cells were washed twice with HBSS and subjected to flow cytometric analysis as described above.

### FerroOrange quantitative analysis

Labile intracellular Fe²⁺ was measured using the fluorescent probe FerroOrange (Sigma-Aldrich, #SC210) and flow cytometry. Following treatment, the medium containing dead cells was removed, and the remaining cells were detached using trypsin, collected, and washed twice with Hank’s Balanced Salt Solution (HBSS). Cells were incubated with 1 µM FerroOrange prepared in serum-free DMEM for 30 min at 37 °C, protected from light. No washing step was performed after staining to prevent dye efflux. After staining, cell suspensions were passed through a 40-µm cell strainer and analyzed immediately by flow cytometry. Fluorescence of FerroOrange was recorded in the PE channel on a BD LSR Fortessa equipped with FACS Diva software (BD Biosciences). Data analysis was performed as described above.

### Western blot

Cells were lysed on ice for 30 min in an ice-cold lysis buffer containing 50 mM Tris-HCl (pH 7.5), 150 mM NaCl, 1% NP-40 (Carl Roth, #8040.3), and 5 mM EDTA, supplemented with PhosSTOP™ (Roche, #4906845001), cOmplete™ protease inhibitor cocktail (Sigma Aldrich, #11836153001), and 1 mM phenylmethylsulfonyl fluoride (PMSF; Sigma Aldrich, #P7626). Cell debris was removed by centrifugation at 14,000 × *g* for 30 min at 4 °C. Following dissection, kidneys were placed on ice to preserve protein integrity. Tissue samples were transferred into 2 ml microcentrifuge tubes, and combined with zirconium oxide beads (Precellys, 10402) and ice-cold lysis buffer (see above) at a ratio of 100 µl buffer per 100 mg of tissue. Homogenization was performed using a Precellys 5500 system with two 20-s pulses. The lysates were then incubated on ice for 30 min. Following incubation, the tubes were centrifuged, and the supernatant was transferred to fresh tubes. Protein concentrations were determined using the BCA assay kit (Thermo Fisher Scientific, #23225) following the manufacturer’s protocol. Equal protein amounts (typically 25 μg per lane) were separated on 4–15% gradient SDS-PAGE gels and transferred onto PVDF membranes (Bio-Rad, #170-4272). Membranes were blocked for 1 h at room temperature, followed by overnight incubation at 4 °C with primary antibodies. Primary antibodies to DPEP-1 (Proteintech, #12222-1-AP, 1:1000), 4-HNE (Abcam, #ab46545, 1:1000), FTH1 (Abcam, #ab65080, 1:1000), GCLM (Abcam, #EPR6667, 1:1000), GCLC (Abcam, #EP13475, 1:2000), ZBP1 (Adipogen, #AG-20B-0010-c100, 1:1000), FSP1 (Santa Cruz, #sc-377120, 1:5000), and β-actin (Cell Signaling, #3700S, 1:500) were diluted in skimmed milk (Carl Roth, #T145.2). Primary antibodies to CD44 (Abcam, #ab189524, 1:30.000), GPX4 (Abcam, #ab125066, 1:5000), GR1 (Cell Signaling/New England Biolabs, #12041, 1:1000), and STING (Cell Signaling, #13647S, 1:1000) were diluted in 5% BSA (Serva, 11930.004).

Secondary HRP-conjugated antibodies against mouse (Cell Signaling, #7076S) and rabbit (Cell Signaling, #7074S) were used at a dilution of 1:5000. Protein detection was performed using enhanced chemiluminescence (ECL; Amersham Biosciences).

Western blot band intensities were quantified with *n* = 3 by densitometry using ImageJ (Fiji) and normalized to the corresponding loading control (β-actin).

### LDH release assay

Lactate dehydrogenase (LDH) release from cells was quantified according to the manufacturer’s protocol (Promega, #G1780). Briefly, at the indicated time points, an aliquot of the culture supernatant was collected to determine experimental LDH release. To assess maximal LDH release, lysis solution was then applied for 45 min, followed by collection of an additional supernatant aliquot. Supernatants were incubated with CytoTox 96® Reagent for 15 min at room temperature, protected from light, after which stop solution was added. Absorbance was measured at 490 nm, and LDH release was calculated using the formula:

$$100\times \left({\rm{experimental\; LDH\; release}}\right)/({\rm{maximum\; LDH\; release}}).$$

### Murine renal ischemia/reperfusion injury (IRI)

Male C57Bl/6N mice were obtained from Charles River Laboratories (Sulzfeld, Germany) at five weeks of age. Animals were maintained under controlled environmental conditions at the Medizinisch-Theoretisches Zentrum (MTZ), Medical Faculty of the Technische Universität Dresden. Housing was provided in individually ventilated cages (IVCs) compliant with at least Euronorm type II standards, with a stable 12-h light/dark cycle. Ambient temperature was kept between 20–24 °C, and relative humidity was maintained between 45–65%, as monitored through daily environmental checks. Sterilized standard pellet diet (Sniff) and autoclaved water were available *ad libitum*. All bedding and enrichment materials were sterilized by autoclaving before use. Mice were group-housed (2–5 animals per cage) and acclimatized until reaching 10 weeks of age prior to surgical procedures.

A detailed protocol for inducing renal ischemia/reperfusion injury has been previously described [15]. Briefly, mice received a single, blinded intraperitoneal injection of either vehicle control (2% DMSO in 0.9% NaCl) or dexamethasone (200 µg/kg in 0.9% NaCl) in a final volume of 200 µl ice-cold solution, administered 30 min prior to surgery. Analgesia was provided through subcutaneous injection of buprenorphine-HCl (Temgesic, Indivior), followed by induction of anesthesia using isoflurane inhalation. Mice were then positioned supine on a heating platform with a closed-loop temperature control system.

A longitudinal abdominal incision was made to access the renal pedicles, which were occluded using micro-serrefine clamps (FST #18055-03) applying 100 g of pressure. The interval between clamping the left and right pedicle was kept strictly below 45 s. Ischemia was sustained for 34 min, after which clamps were removed, and successful reperfusion was visually confirmed. The abdominal wall and skin were then closed using continuous sutures for each layer. To compensate for fluid loss, 1000 µl of pre-warmed Dulbecco PBS was administered intraperitoneally before the mice were returned to IVCs in pairs. After 48 h, blood samples were collected via retro-orbital puncture and analyzed at the clinical chemistry laboratory of the University Hospital Dresden. Following euthanasia, the right kidney was fixed in 4% buffered formalin for a minimum of 24 h before further processing, while the left kidney was snap-frozen in liquid nitrogen and stored for subsequent analyses.

All animal experiments were carried out under strict blinding with respect to both genotype and treatment with small molecules. All procedures complied with German animal welfare regulations and received prior approval from the ethics committees and local authorities in Dresden, including the ethics committee of the Technische Universität Dresden and the Landesdirektion Sachsen (registration number: TVV07/2021).

### Genetically modified mice

The breeding and generation of ZBP1 knockout (ZBP1^−^^/^^−^) mice has been described in detail previously [16], and tissues were kindly provided by Dr. Andreas Linkermann (Universitätsmedizin Mannheim, Universität Heidelberg). Briefly, the *Zbp1* gene was isolated by PCR from genomic DNA extracted from embryonic stem cells (GSI-I). To construct the targeting vector, a 1.6-kb fragment encoding the open reading frame of ZBP1 was replaced with a neomycin resistance cassette and a herpes simplex virus thymidine kinase gene (HSV-TK). The targeting construct was then transfected into embryonic stem cells. Colonies resistant to both G418 and ganciclovir were selected via PCR screening. Homologous recombination events were confirmed by Southern blot analysis, and positive clones were injected into blastocysts derived from C57Bl/6 mice. Heterozygous F1 offspring were interbred to obtain homozygous ZBP1 knockout mice.

The Sting^Gt^ (C57BL/6J-*Sting1*^*gt*^/J) mouse line is a result of a forward genetic screen as described previously [17], and was originally obtained from The Jackson Laboratory. In short, 90 mg/kg of the germ line mutagen N-ethyl-N-nitrosourea (ENU) was applied weekly to C57BL/6 mice over a 3-week period. G3 progeny carrying homozygous recessive mutations were generated by breeding three generations of ENU-treated male mice and screened for their ability to produce type I IFNs. T596A in exon 6 of *Stin*g1 resulted in a lack of type I IFN production in response to *L. monocytogenes*.

### Human renal gene expression data

To compare gene expression data in healthy individuals and patients with AKI, we used an approach published previously of merging scRNA data from KPMP and the Human Kidney Single Cell Transcriptome [18]. These data were analyzed using a tool publicly provided by the Susztak Laboratory (https://susztaklab.com/hk_genemap_kpmp/scRNA; last accessed 19 December 2025).

### Histology

Kidney tissues were processed for histological evaluation by dehydration through a graded ethanol series, followed by clearing in xylene and embedding in paraffin. Sections of 3–5 µm thickness were prepared and stained using periodic acid–Schiff (PAS) reagent following established protocols. Stained slides were examined under an Axio Imager microscope (Zeiss) at magnifications of ×100, ×200, and ×400. Digital images were captured using an AxioCam MR R3 FireWire camera, with image acquisition and analysis performed using Zen 3.9 software (Zeiss). Further image adjustments and processing were conducted using Affinity Photo 2. Assessment of renal tubular damage was performed by an experienced nephropathologist who was blinded to the experimental groups. Tissue damage was graded on a scale from 0 to 10, where 0 indicated no observable injury and 10 represented the highest degree of organ damage.

The scoring criteria focused on key morphological indicators of kidney injury following ischemia-reperfusion, including loss of the brush border, tubular dilation, degeneration, necrosis, and the presence of tubular casts. Each parameter was evaluated using a semi-quantitative scale: absent (0), mild (1–4), moderate (5–6), severe (7–8), and very severe (9–10).

### TUNEL staining

TUNEL staining was performed using the Click-iT Plus TUNEL Assay (Invitrogen) following the manufacturer’s protocol. Briefly, paraffin-embedded tissue sections were deparaffinized, fixed in 4% PFA for 15 min at 37 °C, and subsequently permeabilized with proteinase K. Incorporation of EdUTPs was carried out for 1 h at 37 °C to label sites of DNA fragmentation. This was followed by a copper-catalyzed click reaction using Alexa Fluor™ picolyl azide for 30 min at 37 °C, as per the provided reagents. After washing, nuclei were counterstained with DAPI (5 µg/ml; Invitrogen) for 15 min, followed by three rinses with deionized water. Images were acquired using a Zeiss ApoTome2 system at the Core Facility Cellular Imaging, Technische Universität Dresden, utilizing 405 nm and 488 nm excitation wavelengths. Image analysis was performed with Zen 3.7 software (Zeiss). Quantification of tubular necrosis was conducted by an experienced nephrologist, blinded to the experimental conditions. A representative field was selected, and the number of TUNEL-positive tubules was counted at ×200 magnification.

### Immunofluorescence

Shortly, tissues were fixed, dehydrated and paraffin-embedded in routine conditions. Four-micrometer-thick tissue sections were prepared for immunostaining. Slides were de-paraffinized in two successive baths of xylene and rehydrated in gradually decreasing ethanol baths (95%, 80%, 70% and 50%) for 5 min each, followed by immersion in distilled water. Antigen retrieval was performed by immersing the slides in preheated Universal buffer for 20 min, in a water bath set at +95 °C followed by a cool down phase for 30 min on the bench. Nonspecific antibody binding was prevented by incubating tissues in 2% normal goat serum (Thermo Fisher) for one hour at room temperature. Auto-fluorescence was reduced by incubating the tissue sections in Image-iT FX Signal Enhancer (Thermo Fisher) for 30 min at room temperature. The list of primary and secondary antibodies used in this study is provided below, nuclei were counter-stained using DAPI (2 µg/mL). Images were acquired on a Zeiss Axio Observer 7 and cells were hand-counted in a strictly blinded fashion.

The primary antibodies were as follows: B220 (Rat, 1:200, Biolegend #103202), CD11b (Rabbit, 1:200, Abcam #133357), CD3 (Rabbit, 1:200, Abcam #16669), CD4 (Rat, 1:200, ThermoFisher #14-9766-82), CD8 (Rat, 1:100, ThermoFisher, #14-0808-82), IBA1 (Rabbit, 1:200, VWR #100369-764), and MPO (Rabbit, 1:500, Abcam, #ab208670). Secondary antibodies were anti-Rabbit IgG 488 (Goat, 1:500, Cell Signalling #4412S), 555 (Goat, 1:500, Cell Signalling #4413S), 647 (Goat, 1:500, Cell Signalling #4414S), and 750 (Goat, 1:500, Cell Signalling #4417S) as well as anti-Rat IgG 555 (Goat, 1:500, Cell Signalling #4417S), and 647 (Goat, 1:500, Cell Signalling #4418S).

### Immunohistochemistry

Tissues were fixed, dehydrated and paraffin-embedded in routine conditions. One-micrometer-thick tissue sections were prepared for immunohistochemistry staining. Slides were deparaffinized in two successive baths of xylene and rehydrated in gradually decreasing ethanol baths (2 × 99%, 2 × 96%, 70%, and 40%) for 1 min each, followed by immersion in distilled water. For CCR2 staining, decloaking was performed for 10 min at 110 °C in TRIS/EDTA buffer with 0.05% Tween in a pressure cooker. For 4-HNE staining, no decloaking was performed. Endogenous peroxidases were blocked with 3% H2O2 for 10 min. Nonspecific binding was blocked by immersing the slides in 2% BSA in PBS for 90 min and incubating with background sniper (Biocare, #BS966) for 10 min. Tissue sections were incubated with primary antibodies (4-HNE, abcam, #ab46545, 1:5000, rabbit and CCR2, abcam, ab273050, 1:200, rabbit) diluted in 1% BSA in PBS and incubated at 4 °C overnight. Subsequently, sections were incubated with secondary antibody (anti-rabbit, Cell Signaling, #7074S, 1:200) diluted in 1% BSA for 1 h at room temperature. The staining was developed using the +DAB kit (abcam, ab64238) according to manufacturers’ instructions. Afterwards, slides were counterstained with hematoxylin and dehydrated in increasing ethanol baths followed by xylene according to standard protocol. Images were acquired using a Zeiss ApoTome system at the Core Facility Cellular Imaging, Technische Universität Dresden. Image analysis was performed with Zen 3.7 software (Zeiss). Samples were evaluated in a double-blinded manner. Building on our previous work [9], we scored 4-HNE staining intensity in proximal tubules leveraging 4-HNE staining intensity in adjacent ATN as ferroptotic positive controls. Proximal tubular cell lipid peroxidation was scored semi-quantitatively ranging from 0 (no proximal tubular cell lipid peroxidation, maximal differences in staining intensity of proximal tubules and ATN) to 3 (strong proximal tubular cell lipid peroxidation, no differences in staining intensity of proximal tubules and ATN).

### Additional software

The Graphical abstract was created with BioRender (Tonnus, W. (2026) https://BioRender.com/c0o5fcz). Figures were prepared using Affinity Designer 2.6.0 (Serif) and GraphPad Prism v10.5.0 (GraphPad Software, San Diego, CA, USA).

### Statistics

All statistical evaluations were conducted using GraphPad Prism version 10.5.0 (GraphPad Software, San Diego, CA, USA). For comparisons between two groups, an unpaired Student’s *t* test with Welch’s correction was applied. Quantitative analyses of tissue damage scores and serum concentrations of creatinine and urea were performed using a two-tailed parametric t-test. In addition, paired data without Gaussian distribution were analyzed using the Wilcoxon matched-pairs signed rank test. For dose–response curves, a four-parameter variable slope nonlinear fit model with least-squares regression without weighting was applied. Here, the percentage of annexin V/7-AAD double-negative cells was normalized within each dose and biological replicate to the corresponding vehicle-treated control. For each replicate, values of dexamethasone-treated cells were divided by the matched vehicle value at the same dose. Vehicle was therefore set to 1, and dexamethasone-treated samples are shown as fold change relative to vehicle. For Fig. 4f, statistics were derived from https://susztaklab.com/hk_genemap_kpmp/scRNA.

Results were considered statistically significant at the following thresholds: *p ≤ 0.05, **p ≤ 0.01, ***p ≤ 0.001 or ****p ≤ 0.0001. If no significant difference between groups was detected, we did not indicate this specifically. If not stated otherwise, bar graphs represent the mean + standard deviation (S.D).

## Results

### Dexamethasone exposure reduces late ferroptosis

**Fig. 1:**
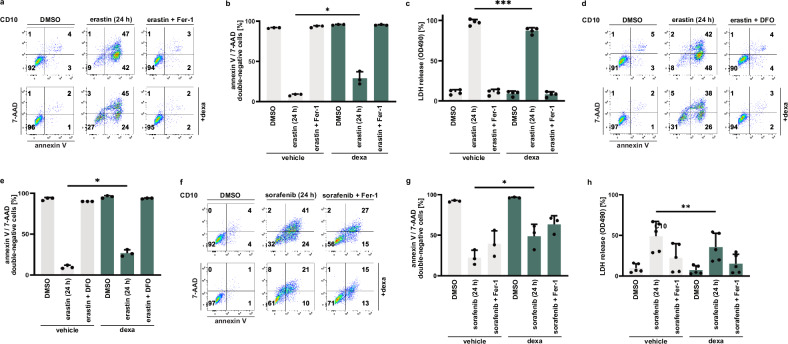
Dexamethasone protects against ferroptosis. CD10-135 cells were pretreated with vehicle solution or 1 µM dexamethasone (“dexa”) for 16 h. **a**, **b** Flow cytometry for annexin V/7-AAD after 24 h of 5 µM erastin treatment. **c** LDH release assay of CD10-135 cells treated with erastin for 24 h. **d**, **e** Flow cytometry for annexin V/7-AAD after 24 h of 5 µM erastin with 50 µM DFO as protection control. **f**, **g** Flow cytometry for annexin V/7-AAD after 24 h of treatment with 10 µM sorafenib. **h** LDH release after 24 h of sorafenib treatment. Data are represented as mean +S.D.

To better understand the effect of dexamethasone on ferroptosis in renal pathophysiology, we first leveraged the human-derived renal tubular cell line CD10-135, which has been demonstrated to recapitulate key features of its origin [14]. We validated that pretreatment with dexamethasone sensitized this cell line to ferroptosis induced by erastin (an inhibitor of system Xc^-^) after 16 h of treatment (Supplementary Fig. S1a, b). However, when we treated CD10-135 cells longer than 16 h with erastin to achieve more efficient ferroptosis induction, we paradoxically observed reduced ferroptosis as quantified by flow cytometry for annexin V/7-AAD staining to detect membrane rupture (Fig. 1a, b). This effect was confirmed using LDH release as a different approach to quantify cell death (Fig. 1c), and by using another inhibitor of ferroptosis (deferoxamine, DFO) as a protection control (Fig. 1d, e). The longer ferroptosis induction also did not lead to increased protein levels of DPEP-1, the proposed mechanism of ferroptosis sensitization by dexamethasone at early timepoints [7] (Supplementary Fig. S1c, d). Mechanistically, we confirmed the specificity of the effect using sorafenib, which is another inhibitor of system Xc^-^, with both methods to detect ferroptosis (Fig. 1f–h). Overall, these data demonstrate that the previous assumption that dexamethasone sensitizes to ferroptosis appears too simplistic and demands further investigations on time- and context-dependent effects.

### Dexamethasone selectively affects ferroptosis stimuli

**Fig. 2:**
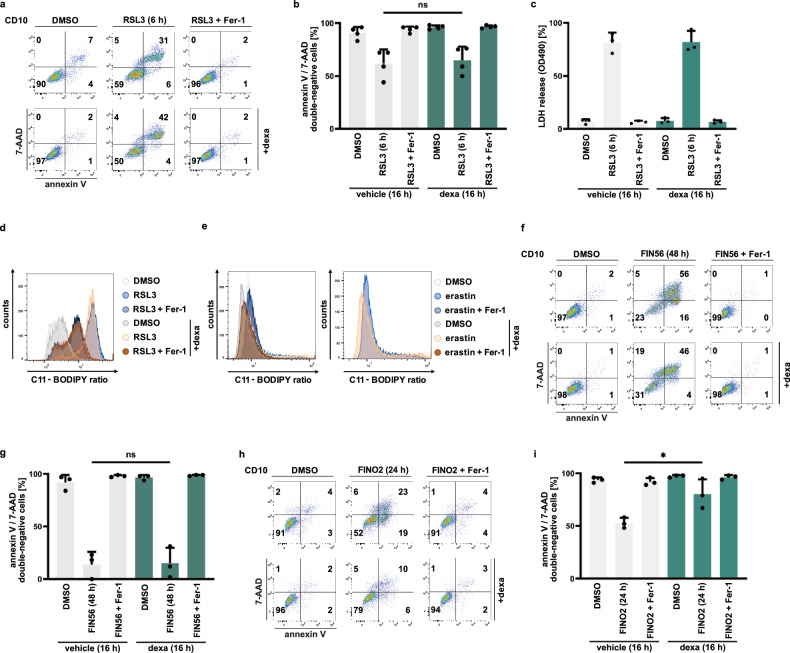
Dexamethasone does not affect all ferroptotic stimuli. CD10-135 cells were pretreated with vehicle solution or 1 µM of dexamethasone for 16 h. **a**, **b** Flow cytometry for annexin V / 7-AAD after 6 h of treatment with 1.13 µM RSL3. **c** LDH release after 6 h of 1.13 µM RSL3. **d** Flow cytometry for C11-BODIPY after 2 h of 1.13 µM RSL3. **e** Flow cytometry for C11-BODIPY after 18 h of 5 µM erastin. **f**, **g** Flow cytometry for annexin V/7-AAD after 48 h of ferroptosis induction with 10 µM FIN56. **h**, **i** Flow cytometry for annexin V/7-AAD after 24 h of ferroptosis induction with 10 µM FINO2. Data are represented as mean +S.D.

To further investigate the mechanism of dexamethasone-dependent ferroptosis modulation, we turned to other ferroptosis-inducing stimuli. As system Xc^-^ is critical to provide cysteine for the biosynthesis of GSH [3], which is required for GPX4 activity [19], we hypothesized that ferroptosis susceptibility upon direct inhibition of GPX4 should not be affected by dexamethasone. Conversely, RSL3 (a covalent inhibitor of GPX4) was equally effective in inducing ferroptosis after pretreatment with either vehicle solution or dexamethasone (Fig. 2a–c). This selectivity of dexamethasone for system Xc^-^ was further supported by quantification of lipid peroxidation, the hallmark of ferroptosis. Whereas C11-BODIPY staining showed no difference in lipid peroxidation induced by RSL3 upon dexamethasone pretreatment (Fig. 2d), reduced lipid peroxidation was seen upon dexamethasone-treatment at later timepoints of ferroptosis induction by erastin (Fig. 2e) or sorafenib (Supplementary Fig. S1e). In line with the biphasic kinetic profile of dexamethasone-mediated ferroptosis modulation, increased accumulation of the non-enzymatic lipoxid protein modification 4-HNE upon erastin treatment was observed (Supplementary Fig. S1f, g).

Furthermore, we tested the class III ferroptosis inducing agent FIN56, which interferes with biosynthesis of CoQ10, a crucial redox equivalent recycled by FSP1 to resist ferroptosis [20]. Again, no significant difference between pretreatment with either vehicle solution or dexamethasone was observed (Fig. 2f, g). As a direct catalysator of ROS generation, the class IV ferroptosis inducer FINO2, however, was less efficient in inducing ferroptosis upon pretreatment with dexamethasone compared to vehicle solution (Fig. 2h, i). These data suggest that, in addition to GR1-dependent depletion of GSH, dexamethasone modulates additional mechanisms of ferroptosis resistance that are independent of both GPX4 and FSP1.

### Anti-ferroptotic effects of dexamethasone are GR1-dependent

**Fig. 3:**
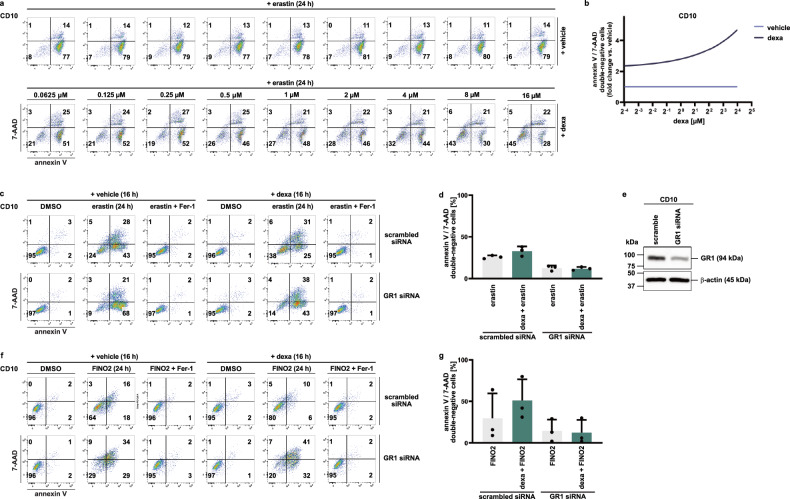
Anti-ferroptotic effects of dexamethasone are GR1-dependent. CD10-135 cells were pretreated with either vehicle solution or dexamethasone for 16 h. **a**, **b** Dose–response curves for dexamethasone upon ferroptosis induction with erastin (5 µM; 24 h) as quantified via flow cytometry for annexin V/7-AAD. **c**, **d** Flow cytometry for annexin V / 7-AAD after erastin-treatment (5 µM; 24 h) in scRNA-treated and GR1 knock-down CD10-135 cells upon pretreatment with either vehicle solution dexamethasone (1 µM) for 16 h. **e** Representative Western blot demonstrating knock-down efficacy of anti-GR siRNA treatment. **f**, **g** Flow cytometry for annexin V/7-AAD after FINO2-treatment (10 µM; 24 h) in scRNA-treated and GR1 knock-down CD10-135 cells upon pretreatment with either vehicle solution dexamethasone (1 µM) for 16 h. Data are represented as mean +S.D.

To gain mechanistic insights into dexamethasone-mediated ferroptosis resistance, we first performed dose–response studies. As expected, escalating doses of dexamethasone did not affect RSL3-induced ferroptosis in CD10 cells (Supplementary Fig. S2a, b). However, upon ferroptosis induction with erastin, a clear dose-dependency of dexamethasone was observed (Fig. 3a, b). Taken together, these results largely rule out direct radical trapping activity of dexamethasone but rather imply involvement of the cognate receptor, GR1. Conversely, dexamethasone failed to reduce erastin-induced ferroptosis in CD10 cells (Fig. 3c, d) upon siRNA-mediated knock-down of the GR1 (Fig. 3e). Furthermore, protective effects of dexamethasone upon FINO2-induced ferroptosis were as well abolished by GR1 knock-down (Fig. 3f, g). In line with previous reports [7], these data demonstrate that dexamethasone effects on ferroptosis are dependent on the GR1. Therefore, we set out to investigate downstream mechanisms mediating ferroptosis resistance.

### Dexamethasone alters iron handling

**Fig. 4:**
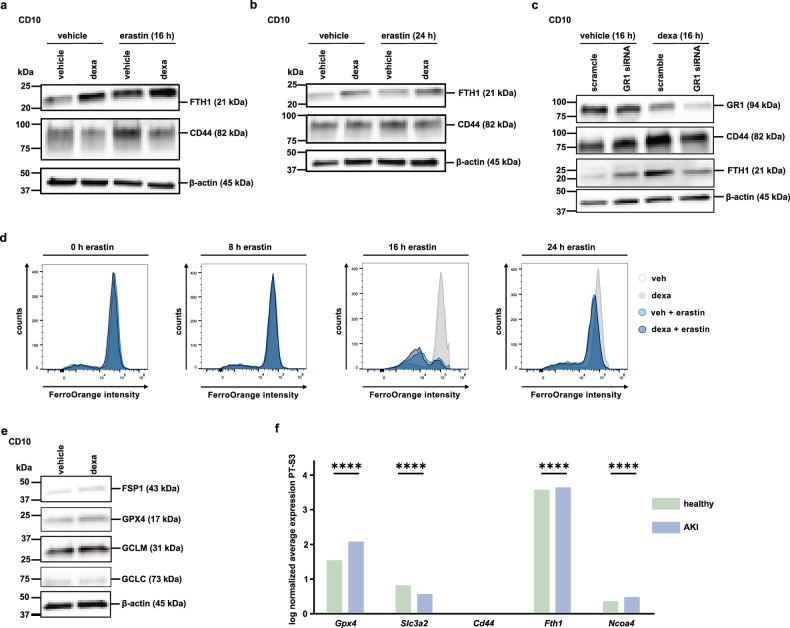
Dexamethasone alters iron handling. CD10-135 cells were pretreated with vehicle solution or 1 µM dexamethasone for 16 h. **a** Western blot of FTH and CD44 after 16 h of treatment with vehicle or 5 µM erastin. **b** Western blot of FTH and CD44 after 24 h of treatment with vehicle or 5 µM erastin. **c** Representative Western blot for GR1, CD44, and FTH1 after scRNA- or siRNA-treatment to GR1. **d** Representative flow cytometry for FerroOrange staining intensity over time of erastin treatment. **e** Western blot for protein levels of GCLM, GCLC, FSP1, and GPX4. **f** Human gene expression data of healthy participants and patients with AKI.

Recently, system Xc^-^ has been described as a non-canonical regulator of lysosomal pH [21], which affects availability of the labile iron pool and implies erastin to increase ferrous iron (Fe^2+^) levels. As FINO2 also requires Fe^2+^ to catalyze ROS generation, we next investigated if how dexamethasone reduces ferroptosis by affecting lysosomal iron availability. Ferric iron (Fe^3+^) is stored within ferritin in the cytoplasm, whereas Fe^2+^ for ferroptosis induction is either imported via CD44 from the extracellular compartment [22] or provided via lysosomal ferritin degradation and subsequent reduction [23]. In line with its ferroptosis-reducing effects, protein levels of FTH1 (ferritin heavy chain 1) were increased in a dexamethasone-dependent manner both in absence of erastin as well as upon short and long erastin exposure (Fig. 4a, b, Supplementary Fig. S3a, b). As previously reported [22], CD44 levels were increased upon ferroptosis induction reflecting altered lysosomal iron dynamics (Fig. 4a, Supplementary Fig. S3b). Opposingly, dexamethasone diminished CD44 upregulation only at early timepoints of erastin exposure implying to rather mirror functional changes than direct effects (Fig. 4a, b, Supplementary Fig. S3c, d). In line with this hypothesis, GR1 knock-down attenuated FTH1 upregulation upon dexamethasone pretreatment, but did not increase CD44 expression in combination with dexamethasone (Fig. 4c, Supplementary Fig. S3e–g). Functionally, these findings correlated with reduced iron availability upon dexamethasone pretreatment after 24 h of erastin treatment as assessed by flow cytometry for FerroOrange staining (Fig. 4d). Conversely, protein levels of GCLM/GCLC (required for GSH biosynthesis), GPX4, and FSP1 were unaffected by dexamethasone pretreatment (Fig. 4e, Supplementary Fig. S3h). Overall, these data point to complex modulation of ferroptosis by dexamethasone: Whereas reduced GSH availability sensitizes to ferroptosis at earlier timepoints as demonstrated previously, reduced iron availability appears to reduce ferroptosis at later timepoints.

### Dexamethasone reduces acute tubular necrosis without improving renal failure

**Fig. 5:**
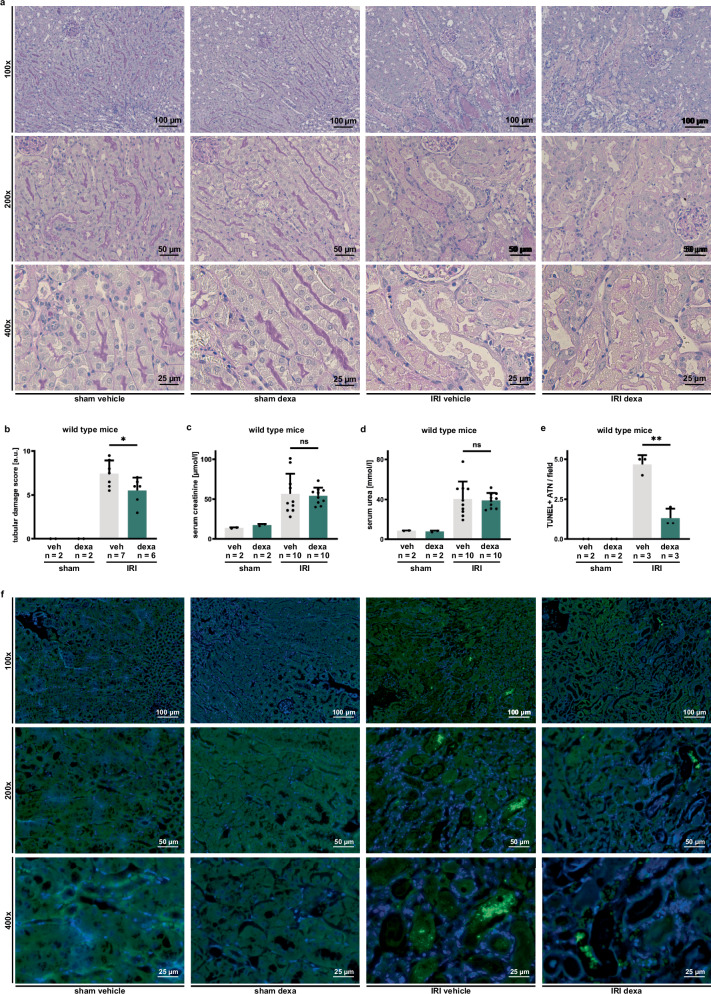
Dexamethasone reduces tubular injury upon AKI. 8–12 weeks-old male C57Bl/6N mice underwent bilateral renal ischemia followed by 48 hours of reperfusion injury before sacrifice. **a** Representative micrographs of PAS-stained renal sections. **b** Quantification of tubular injury by an established score. **c**, **d** Values of serum creatinine and urea. **e** Quantification of renal tubular cell death via TUNEL staining. **f** Representative micrographs of immunofluorescence for TUNEL (green) and DAPI (blue). Data are represented as mean +S.D.

To understand how these opposing mechanisms affect ferroptosis in complex pathophysiological settings, we next focused on acute kidney injury (AKI), which has been established as a standard model for ferroptotic cell death in vivo [9]. Single-cell gene expression analysis from a publicly available database [18] suggested dysregulation of both GPX4 as well as iron handling in proximal tubular epithelial cells of the S3 segments, which are known to be the most ferroptosis-prone part of the nephron[24] (Fig. 4f). Ischemia/reperfusion injury (IRI) is a typical murine AKI model [15]. 48 h after reperfusion, analysis of PAS-stained renal slides revealed reduced tubular injury in dexamethasone-treated animals compared to vehicle-treated littermates (Fig. 5a, b). Although renal function as assessed by serum values of creatinine and urea was not improved (Fig. 5c, d), TUNEL staining again demonstrated significantly reduced renal tubular cell death upon dexamethasone pretreatment (Fig. 5e, f, Supplementary Fig. S4a). Conversely, pretreatment with dexamethasone reduced lipid peroxidation in non-ferroptotic renal tubules as quantified by IHC for 4-HNE (Supplementary Fig. S4b, c). Overall, these data were well in line with the increased ferroptosis resistance conferred by dexamethasone in cell culture, so we speculated that non-ferroptotic effects of dexamethasone might be responsible for the lack of functional renal protection.

### Dexamethasone alters immune cell infiltration upon AKI

**Fig. 6:**
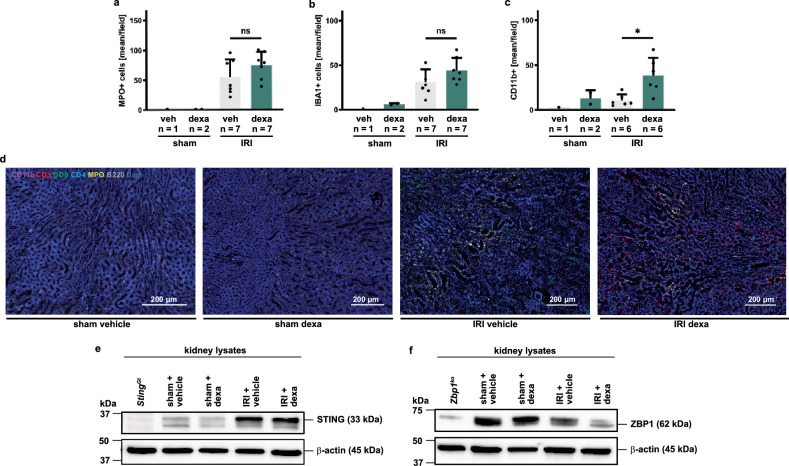
Dexamethasone alters necroinflammation in AKI. 8–12 weeks-old male C57Bl/6N mice underwent bilateral renal ischemia followed by 48 h of reperfusion injury before sacrifice. **a**–**c** Quantification of MPO+ (neutrophils), IBA1+ (pan-macrophage), and CD11b+ (pan-myeloid) cells in renal sections after IRI. **d** Representative merged micrographs of renal sections for CD11b, CD3, CD8, CD4, MPO, B220 and DAPI. **e** Western blot of whole kidney lysates for protein levels of STING. **f** Western blot of whole kidney lysates for protein levels of ZBP1. Data are represented as mean + S.D.

In addition to the cell death itself, the subsequent inflammatory response (“necroinflammation”) is an important factor determining renal function [2]. As dexamethasone has broad immunosuppressive effects including modulation of myeloid cell functions [25], we speculated that alterations in necroinflammation might explain this discrepancy. To this end, we applied a multiplex-based immunofluorescence approach to assess immune cell populations on the same tissue section. As expected, cells of the adaptive immune system were rare, whereas innate immune cell populations were abundant (Supplementary Fig. S5a, b). No significant differences were detected for neutrophils (MPO+, Fig. 6a) or macrophages (IBA1+, Fig. 6b) but for the pan-myeloid marker CD11b (Fig.6c). These CD11b+ cells were in close spatial relation to necrotic casts (Fig. 6d, Supplementary Fig. S5a, b), indeed suggesting an important role in necroinflammation. Importantly, we could not detect relevant amounts of CCR2+ cells (C-C chemokine receptor type 2, a typical marker of infiltrating monocytes and some neutrophil subsets [26]) although having established solid positive controls (Supplementary Fig. S5c). These data demonstrate that high-dose glucocorticoids complexly alter innate immune cell composition upon IRI.

In an additional approach to gain insights into immune cell infiltration, we investigated the expression of typical proteins in whole kidney lysates. Western blot for STING demonstrated strong increases upon IRI, but STING expression was unaffected by dexamethasone pre-treatment (Fig. 6e). ZBP1 expression, however, was lower upon IRI. This effect was further enhanced by dexamethasone pre-treatment (Fig. 6f). As both proteins are highly specific for infiltrating immune cells [18], these data again demonstrate that dexamethasone selectively alters the composition of infiltrating myeloid cells.

## Discussion

Treatment with high-dose glucocorticoids remains a mainstay upon solid organ transplantation to prevent acute rejection [8]. As ferroptosis has been identified as a pathophysiological hallmark of IRI upon transplantation [4], this work aimed to investigate how high-dose glucocorticoids affect ferroptosis and, importantly, subsequent organ function in the typical model of renal IRI [15].

First, we probed the prevailing concept that dexamethasone generally sensitizes to ferroptosis.

We confirmed that dexamethasone increased ferroptosis susceptibility at early timepoints, and observed accumulation of 4-HNE modification as proxy for lipid peroxidation with erastin upon dexamethasone pretreatment, which is in line with GR1-dependent reduction of GSH [7]. However, our data demonstrate a biphasic kinetic profile of dexamethasone-mediated ferroptosis modulation with protective effects upon longer exposure of renal epithelial cells to pro-ferroptotic stimuli.

Mechanistically, we found that the early pro-ferroptotic effect of dexamethasone, mediated through system Xc⁻–dependent impairment of GSH biosynthesis [7], was subsequently overridden by reduced iron availability at later time points. Dexamethasone quenched the labile iron pool by fostering an GR1-dependent increase in iron storage within ferritin, which also correlated to a reduced expression of the lysosomal iron import protein CD44. Previous reports suggested that system Xc^-^ is involved in lysosomal pH regulation [21], but this study for the first time demonstrates direct relevance of this mechanism for iron handling and ferroptosis. This was further evidenced by the recapitulation of anti-ferroptotic effects upon treatment with sorafenib, another inhibitor of system Xc^-^. Importantly, it was previously reported that sorafenib requires ferrous iron to efficiently exert its anti-tumor effects [27]. In line with this concept - other than system Xc- inhibitors - dexamethasone only affected ferroptosis induction via FINO2, a drug which iron-dependently generates ROS, but not with other canonical ferroptosis inducers such as direct GPX4 inhibitors.

Having laid the foundation by improving understanding of glucocorticoid-dependent ferroptosis modulation in proximal tubular renal epithelial cells, we then aimed to investigate how these observations translate to the complex pathophysiology of renal transplantation. Our setting featuring pretreatment with high-dose glucocorticoids followed by warm IRI closely mimics this clinical situation. Importantly, it was previously reported that dexamethasone sensitizes isolated renal tubules to spontaneous ferroptosis [7]. Yet, we observed that dexamethasone treatment led to reduced tubular injury in vivo, demonstrating the importance of the iron-dependent anti-ferroptotic dexamethasone effects. This observation is consistent with previous reports demonstrating protection from renal ischemia–reperfusion injury conferred by sorafenib [28]. Furthermore, these data contribute to understand why some ferroptosis-targeting strategies are highly efficient to reduce ATN [9, 29], whereas others highlight a disconnect between potent ferroptosis-modulating effects observed in cell culture and the comparatively modest impact of such interventions in AKI in vivo, exemplified by FSP1 deficiency [6, 9, 10].

However, we observed that reduced tubular cell death did not translate into improved renal function following AKI, as these effects were countered by dexamethasone-mediated alterations in innate immune cell recruitment. This finding is in line with previous reports on bidirectional effects of glucocorticoids on innate immune cells [30]. Furthermore, it both underscores the pathophysiological importance of both components of necroinflammation - regulated cell death and inflammation - in shaping disease outcomes as well as highlights their complex interplay. Specifically, we observed a marked accumulation of CD11b+ cells (a pan-myeloid marker) in close spatiotemporal relation to ATN. However, we did neither observe a significant increase in MPO+ (neutrophils) nor IBA1+ (classical macrophages) cells. IHC for CCR2 (infiltrating monocytes) also did not demonstrate marked increases, which currently leaves the exact identity of these CD11b+ cells open. Typically, CD11b would also be expressed on immature myeloid progenitor cells or dendritic cell subsets. Future studies should aim to further characterize these cells, how they contribute to renal injury, and how they are both modulated by and interact with ongoing proximal tubular cell ferroptosis.

Despite these exciting results, some caveats for interpretation of this study should be mentioned. Naturally, the complexity of a murine model might involve additional effects of glucocorticoids for which we cannot fully control. These could include, but are not necessarily limited to, glucocorticoid-induced alterations in hemodynamics, sympathetic activity, or glucose metabolism. Furthermore, our studies were limited to male mice – if these findings apply to females, which have been demonstrated to be inherently resistant to ferroptosis upon IRI [6], should be addressed in future. Regarding other ferroptosis-regulating systems than iron metabolism, we cannot exclude that glucocorticoids also affect the lipidome, which might contribute to the phenotype observed.

In summary, our study identifies glucocorticoid-driven iron sequestration as a potentially important link between ferroptotic tissue injury and the inflammatory response in renal IRI. In this context, dexamethasone-induced iron storage may reduce the labile iron pool, thereby limiting lipid peroxidation and ferroptotic damage. More broadly, these findings place our work at the intersection of kidney injury, nutritional immunity, and disease tolerance: iron redistribution during inflammation is increasingly recognized not only as a mechanism to restrict iron availability, but also as a strategy to preserve tissue integrity under stress. Thus, the effects observed here may extend beyond solid organ transplantation and could be relevant to other clinical settings associated with a high risk of acute kidney injury and exposure to high-dose glucocorticoids, including severe systemic inflammatory states (e.g., for COVID19 pneumonia [31]). Generally, our findings argue that the interaction between glucocorticoid signaling and systemic iron handling warrants greater consideration in both experimental and clinical settings.

Overall, this study positions ferroptosis as a temporally dynamic and context-dependent process within the complex landscape of ATN, rather than a static, cell-autonomous death program. Our findings highlight necroinflammation as a critical integrative framework through which regulated cell death and immune responses jointly determine tissue injury and functional outcome. A deeper mechanistic understanding of how systemic signals modulate ferroptosis-immune crosstalk in vivo may enable the development of therapeutic strategies that precisely recalibrate necroinflammatory pathways, thereby delineating how targeted modulation of necroinflammatory pathways can be leveraged to uncouple cell death from adverse immune effects and thereby refine future therapeutic strategies.

## Supplementary information

Supplementary figures

uncropped Western blots

## Data Availability

Human renal gene expression data were obtained by merging scRNA data from KPMP and the Human Kidney Single Cell Transcriptome using a tool publicly provided by the Susztak Lab (https://susztaklab.com/hk_genemap_kpmp/scRNA). Source data are provided. Uncropped Western blots are provided in the Supplementary Information. Materials and additional information are available upon reasonable request via the corresponding author.
