# Supplementary material for: Remodeling of ferroptotic necroinflammation by dexamethasone in acute tubular necrosis
Source: Cell Death Dis. 2026 Jul 15;17(1):644. doi: 10.1038/s41419-026-09099-w (PMC13373221; doi:10.1038/s41419-026-09099-w)
Supplement: Supplementary file 1 — Supplementary figures [file 41419_2026_9099_MOESM1_ESM.docx]

**Supplementary information to:**

**Remodeling of ferroptotic necroinflammation by dexamethasone in acute tubular necrosis**

Natalie Bethe^1,^*, Mirela Tmava^1,^*, Alix Bruneau^2^, Karolin Flade^1^, Benjamin Böhme^1^, Erik Klapproth^3^, Selina Michel^1^, Marlena Nastassja Schlecht^1^, Siyu Xu^1^, Ali El-Armouche^3^, Frank Tacke^2^, Stefan R. Bornstein^1,4^, and Wulf Tonnus^1,5^


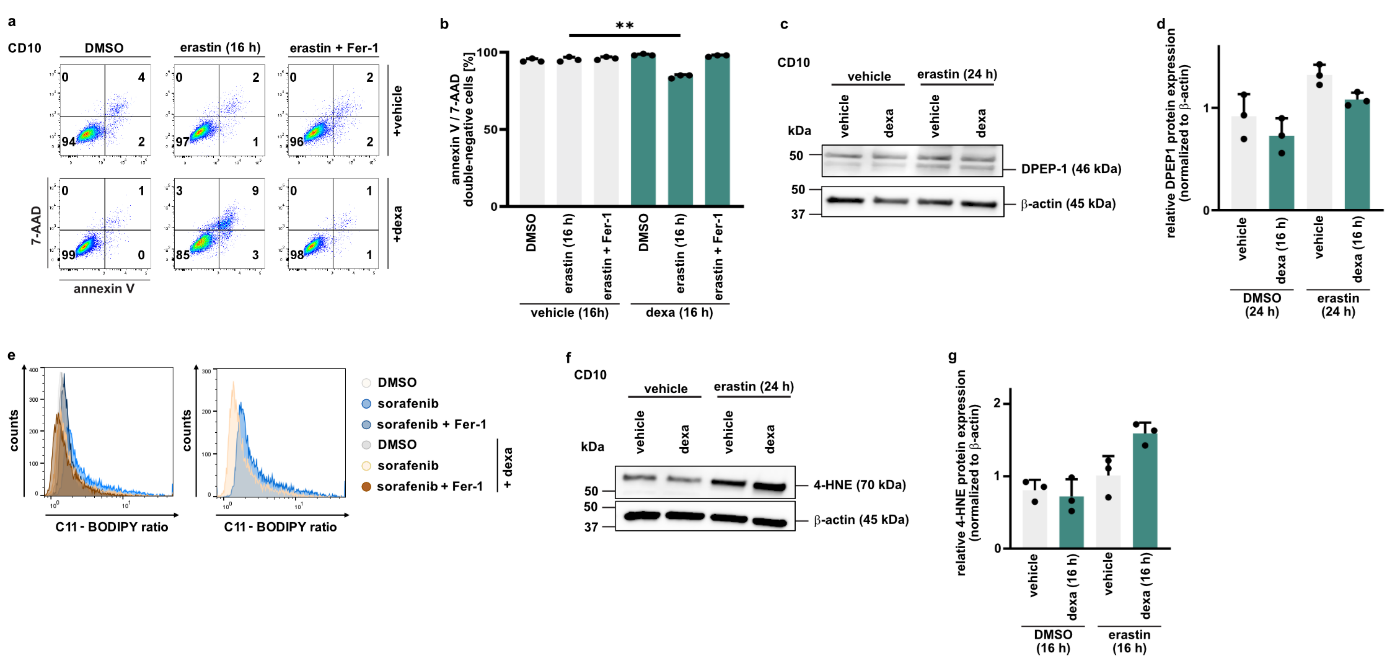


**Figure S1: Dexamethasone sensitizes to ferroptosis at early timepoints**

CD10-135 cells were pretreated with vehicle solution or 1 µM dexamethasone (dexa) for 16 hours. (**a-b**) Flow cytometry for annexin V / 7-AAD after 16 hours treatment with 5 µM erastin. (**c**) Representative Western blot for DPEP-1 protein levels. (**d**) Quantification of protein expression of DPEP-1 as presented in Western blot **Fig. S1c**. (**e**) Flow cytometry for C11-BODIPY after 21 hours of 10 µM sorafenib. (**f**) Western blot for 4-HNE after 24 hours of 5 µM erastin. (**g**) Quantification of protein expression of 4-HNE.


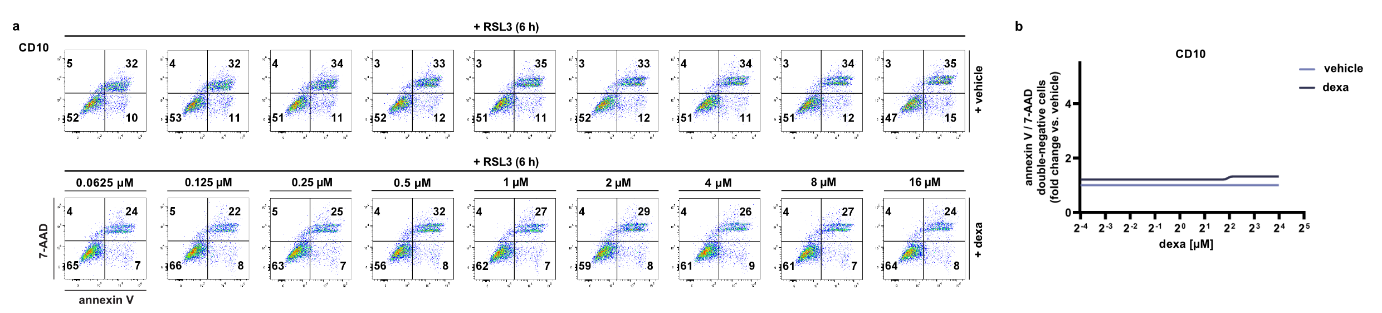


**Figure S2: GR1 is not involved in ferroptosis upon GPX4 inhibition**

CD10-135 cells were pretreated with either vehicle solution or dexamethasone for 16 hours. (**a-b**) Dose-response curves for dexamethasone upon ferroptosis induction with RSL3 (1.13 µM; 6 hours) as quantified via flow cytometry for annexin V / 7-AAD.

**
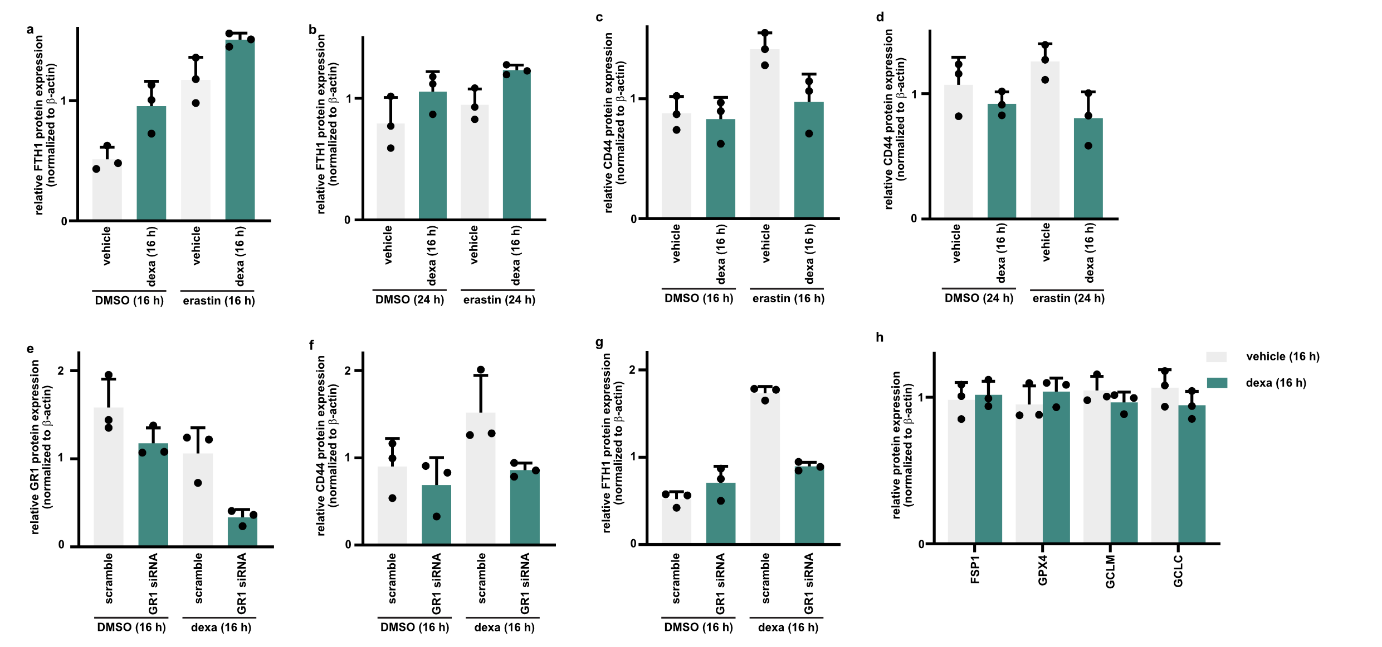
**

**Figure S3: Dexamethasone alters iron handling**

Protein expression as deduced from representative Western blots were quantified as normalized to β-actin. Quantification of (**a**-**b**) FTH expression and (**c-d**) CD44 expression corresponding to **Figure 4a-b**. (**e-g**) Quantification of GR1, CD44, and FTH1 expression in scRNA-treated vs GR1 knock-down CD10 cells corresponding to **Figure 4c**. (**h**) Quantification of FSP1, GPX4, GCLM, and GCLC expression corresponding **to Figure 4e**.

**
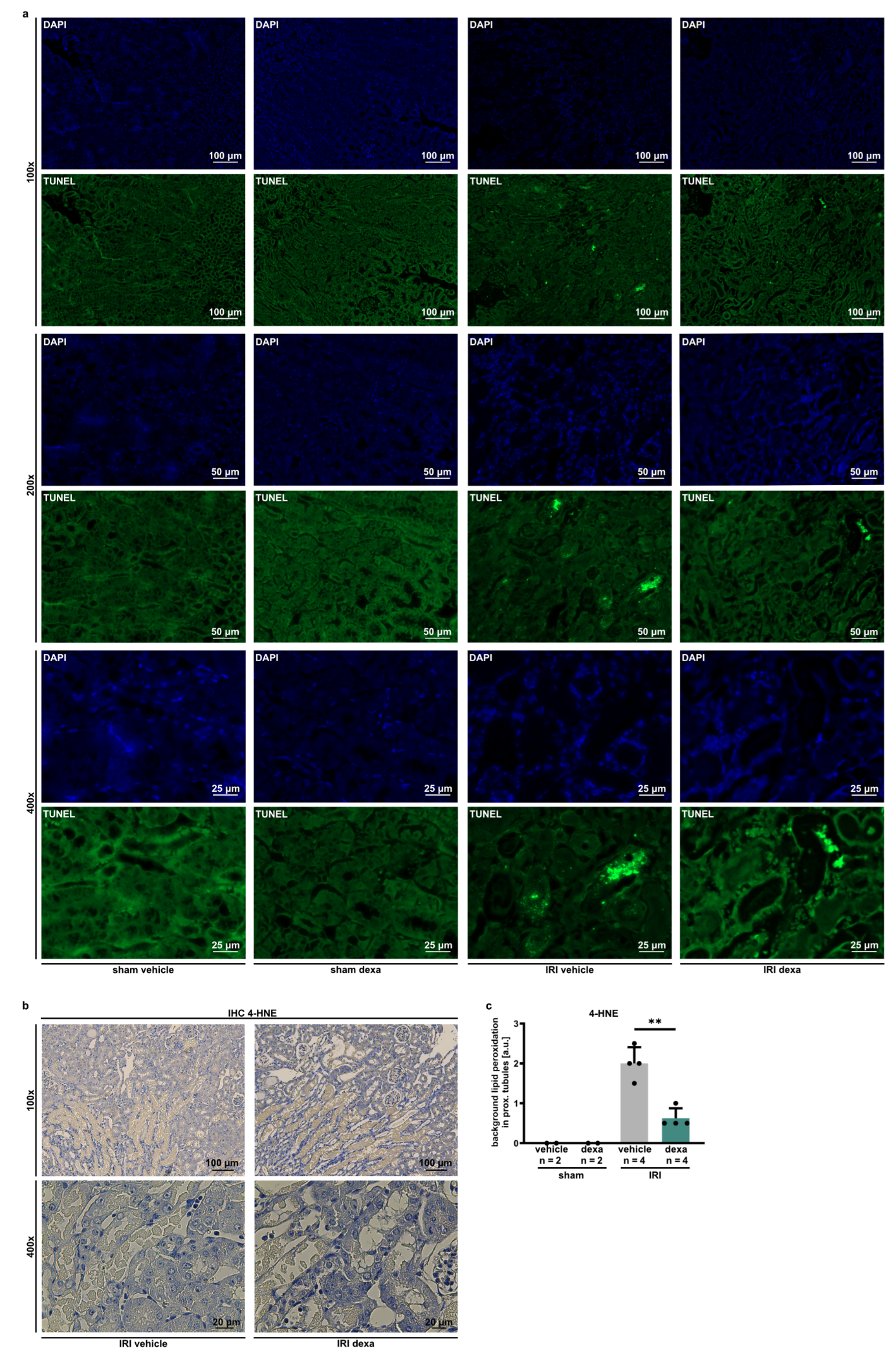
**

**Figure S4: Dexamethasone reduces tubular cell necrosis**

(**a**) Representative single-channel micrographs upon immunofluorescence for TUNEL (green) and DAPI (blue). (**b-c**) Quantification of renal tubular lipid peroxidation by IHC for 4-HNE 48 hours after IRI.

**
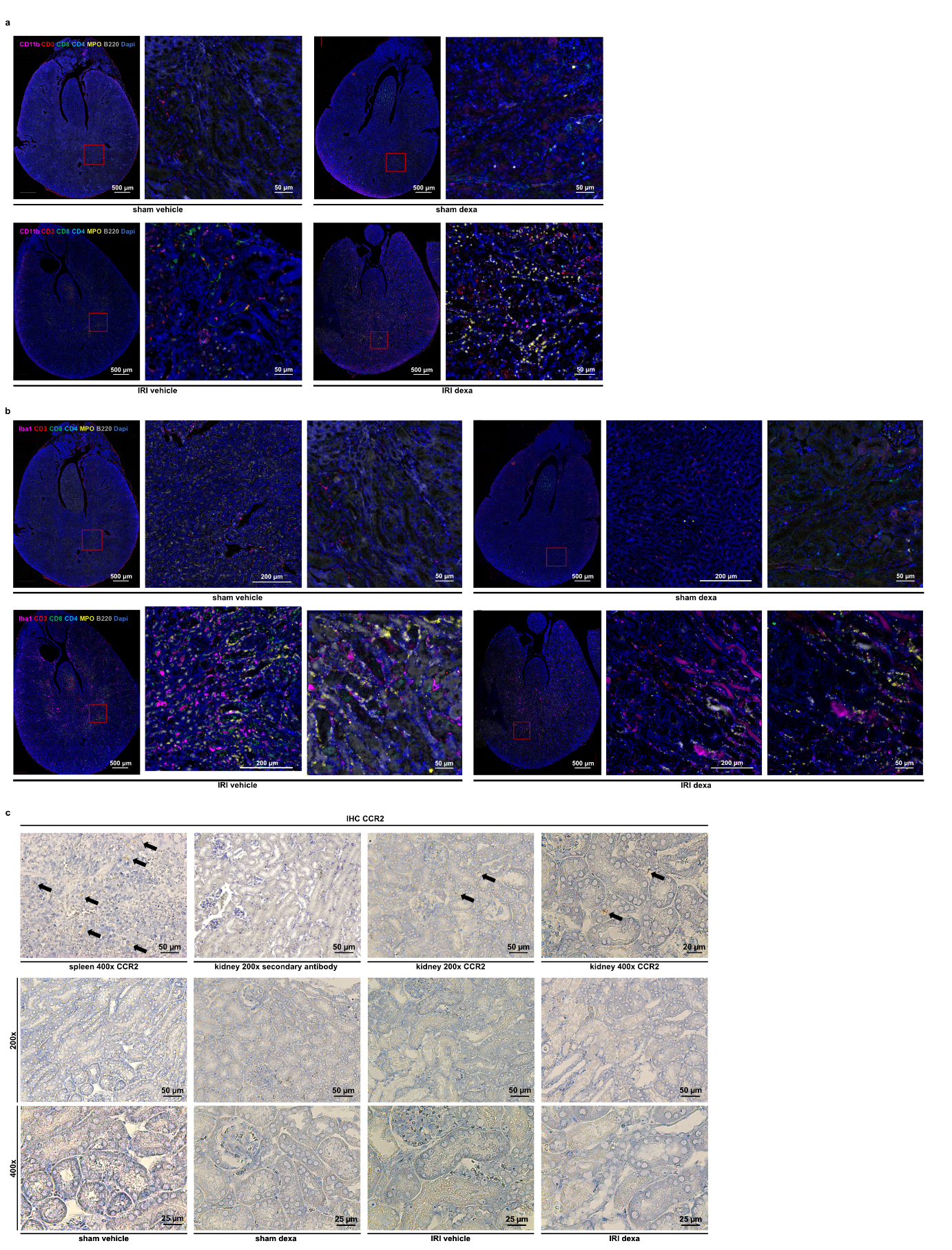
**

**Figure S5: Dexamethasone alters immune cell composition upon IRI**

8-12 weeks-old male C57Bl/6N mice underwent bilateral renal ischemia followed by 48 hours of reperfusion injury before sacrifice. (**a**) Representative merged micrographs at different magnifications of renal sections for CD11b, CD3, CD8, CD4, MPO, B220 and DAPI. (**b**) Representative merged micrographs at different magnifications of renal sections for IBA1, CD3, CD8, CD4, MPO, B220 and DAPI. (**c**) Representative micrographs of IHC for CCR2. The upper lane shows micrographs of the controls establishing specificity for CCR2 staining with detectable CCR2+ cells in spleen tissue and selected samples after IRI (arrows). Lower lane shows no relevant CCR2+ cells throughout investigated groups.
