# Supplementary material for: Remodeling of ferroptotic necroinflammation by dexamethasone in acute tubular necrosis
Source: Cell Death Dis. 2026 Jul 15;17(1):644. doi: 10.1038/s41419-026-09099-w (PMC13373221; doi:10.1038/s41419-026-09099-w)
Supplement: Supplementary file 2 — uncropped Western blots [file 41419_2026_9099_MOESM2_ESM.pptx]

## Slide 1
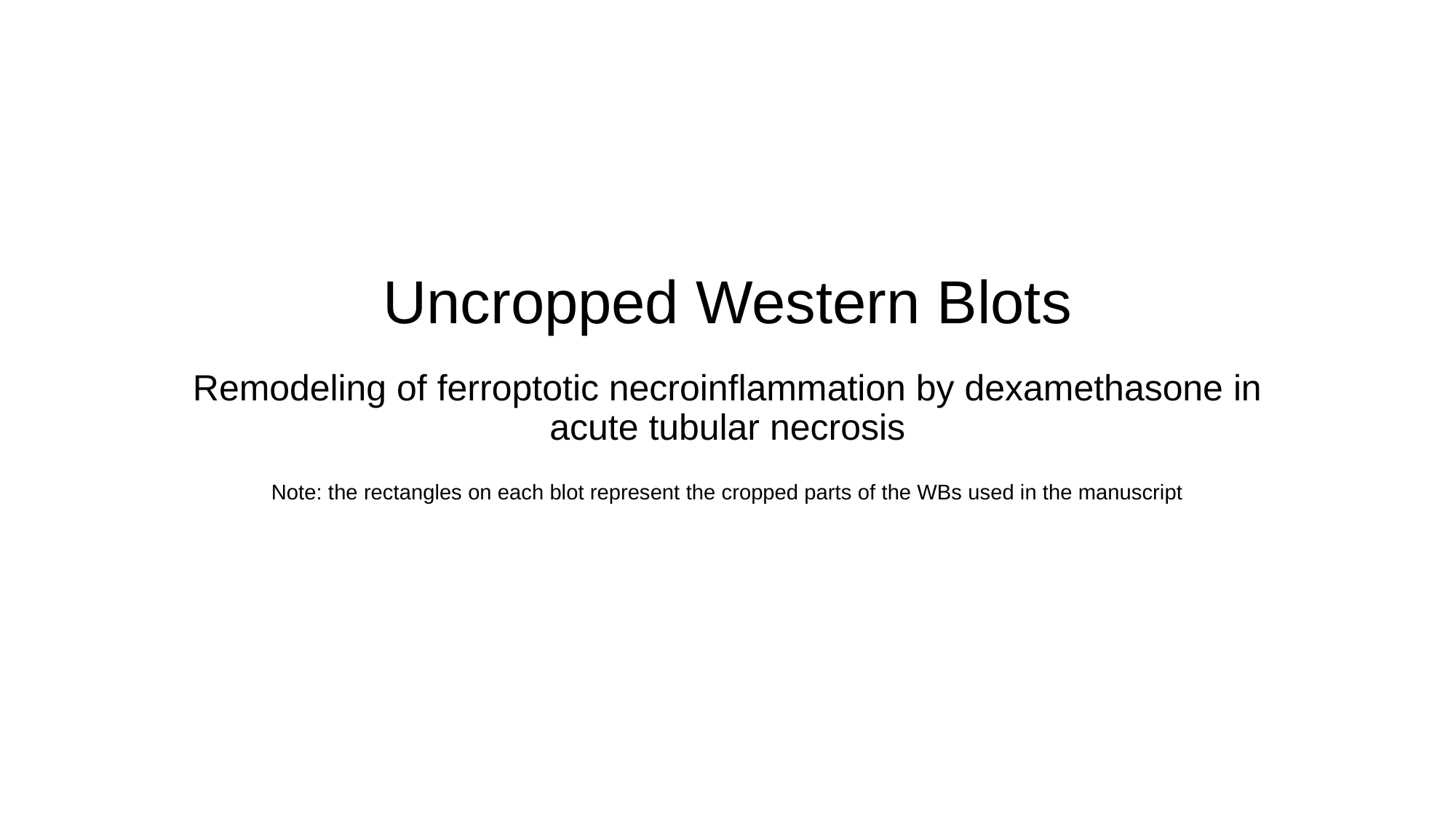

# Uncropped Western Blots
Remodeling of ferroptotic necroinflammation by dexamethasone in acute tubular necrosis
Note: the rectangles on each blot represent the cropped parts of the WBs used in the manuscript

## Slide 2
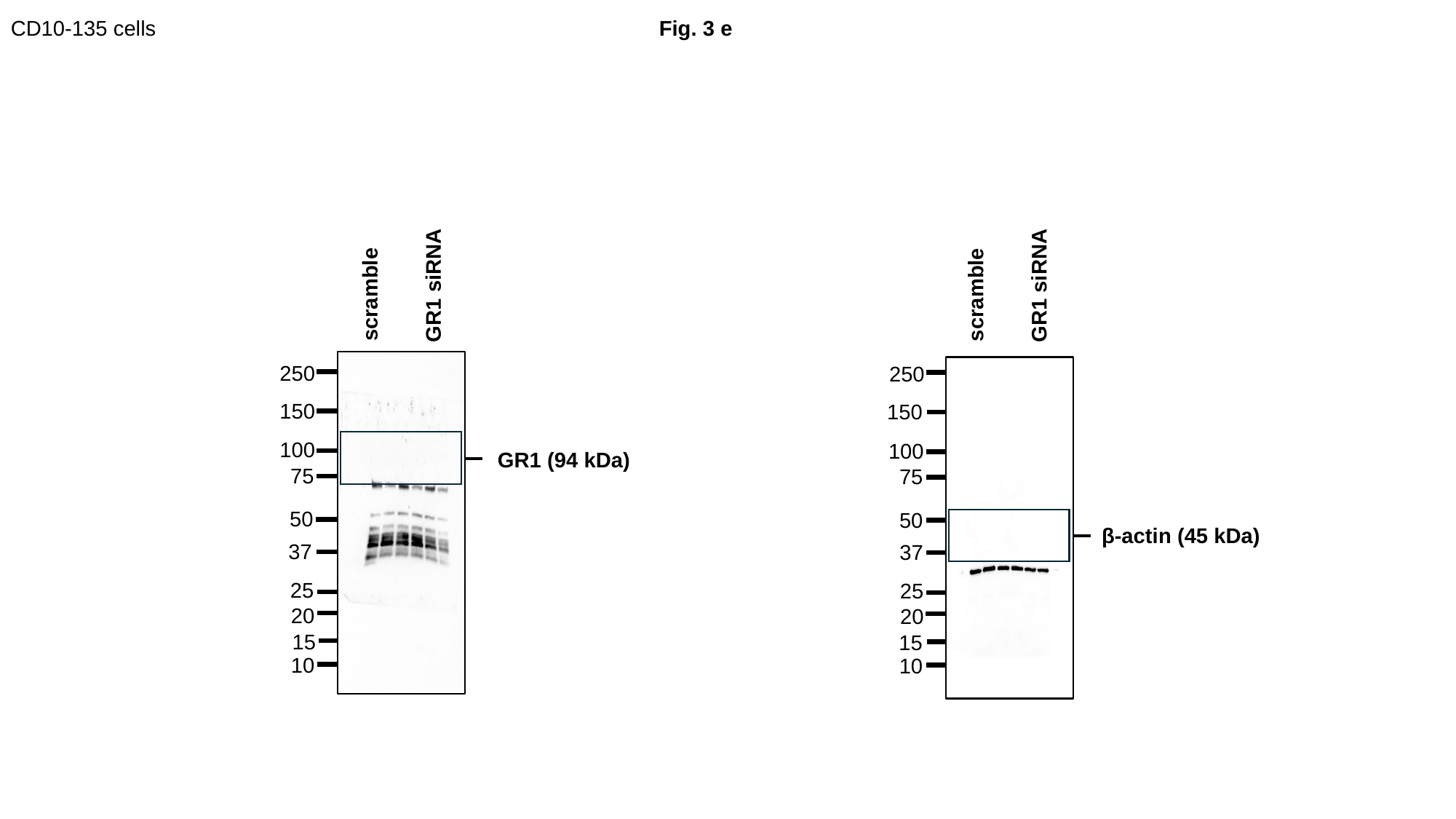

CD10-135 cells
Fig. 3 e
scramble
scramble
GR1 siRNA
GR1 siRNA
250
250
150
150
100
100
GR1 (94 kDa)
75
75
50
50
β-actin (45 kDa)
37
37
25
25
20
20
15
15
10
10

## Slide 3
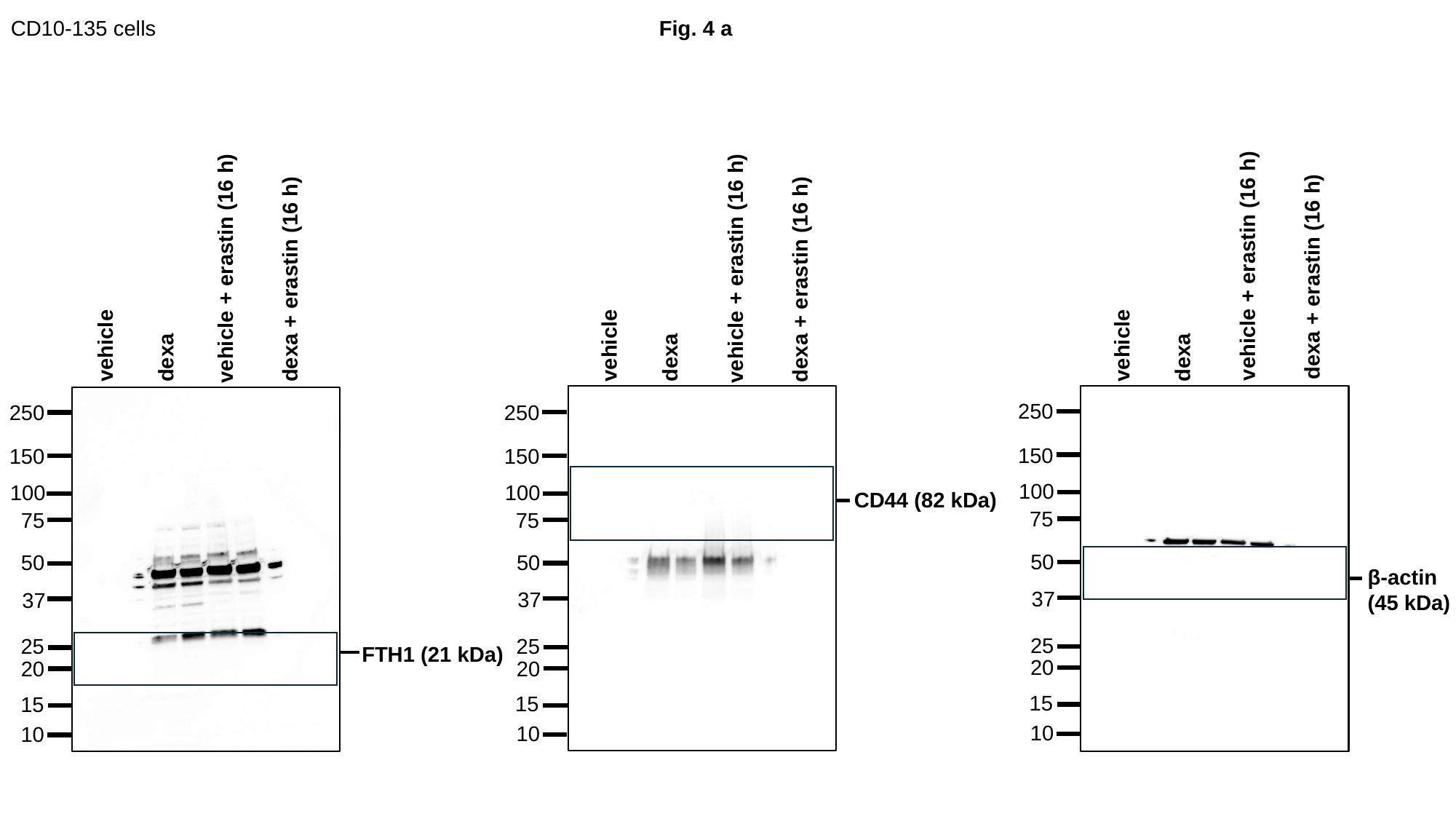

CD10-135 cells
Fig. 4 a
dexa + erastin (16 h)
vehicle + erastin (16 h)
dexa + erastin (16 h)
dexa + erastin (16 h)
vehicle + erastin (16 h)
vehicle + erastin (16 h)
dexa
dexa
dexa
vehicle
vehicle
vehicle
250
250
250
150
150
150
100
100
100
CD44 (82 kDa)
75
75
75
50
50
50
β-actin
(45 kDa)
37
37
37
25
25
25
FTH1 (21 kDa)
20
20
20
15
15
15
10
10
10

## Slide 4
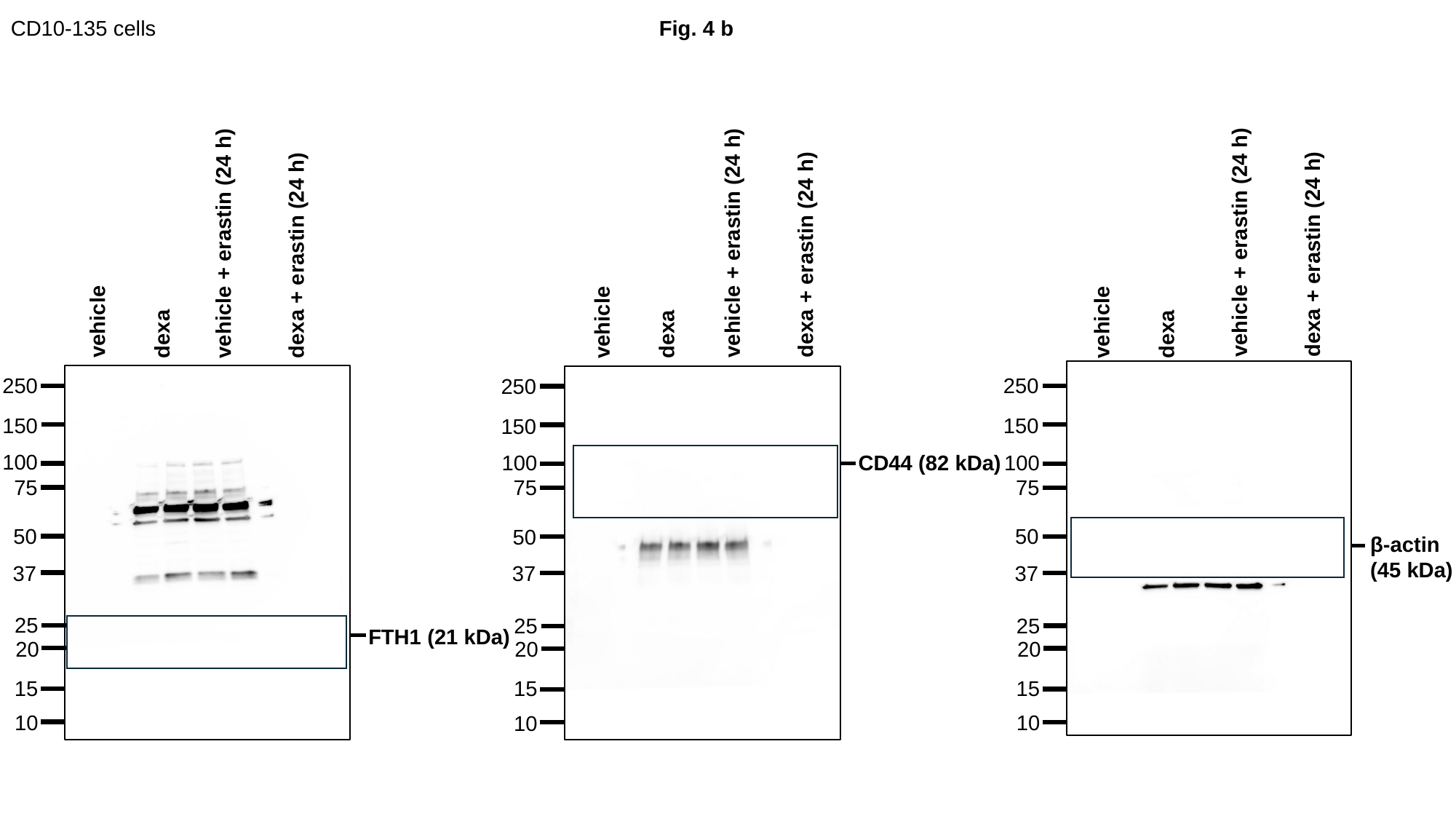

CD10-135 cells
Fig. 4 b
vehicle + erastin (24 h)
dexa + erastin (24 h)
vehicle + erastin (24 h)
dexa + erastin (24 h)
dexa
vehicle + erastin (24 h)
dexa + erastin (24 h)
dexa
dexa
vehicle
vehicle
vehicle
250
250
250
150
150
150
100
100
100
CD44 (82 kDa)
75
75
75
50
50
50
β-actin
(45 kDa)
37
37
37
25
25
25
FTH1 (21 kDa)
20
20
20
15
15
15
10
10
10

## Slide 5
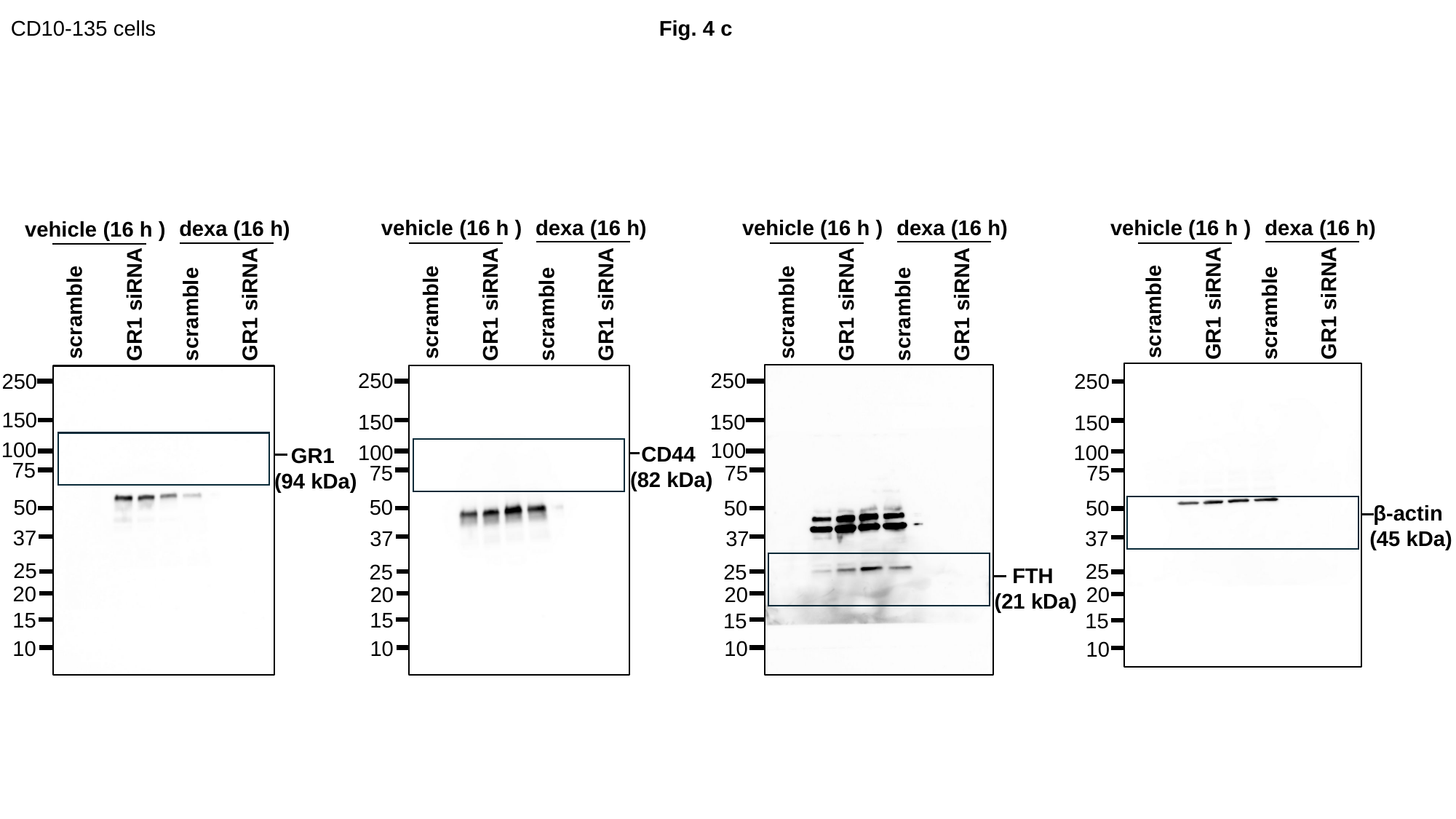

CD10-135 cells
Fig. 4 c
dexa (16 h)
dexa (16 h)
dexa (16 h)
vehicle (16 h )
vehicle (16 h )
vehicle (16 h )
dexa (16 h)
vehicle (16 h )
GR1 siRNA
GR1 siRNA
GR1 siRNA
GR1 siRNA
GR1 siRNA
GR1 siRNA
GR1 siRNA
GR1 siRNA
scramble
scramble
scramble
scramble
scramble
scramble
scramble
scramble
250
250
250
250
150
150
150
150
100
100
100
100
CD44
(82 kDa)
GR1
(94 kDa)
75
75
75
75
50
50
50
50
β-actin
(45 kDa)
37
37
37
37
25
25
25
25
FTH
(21 kDa)
20
20
20
20
15
15
15
15
10
10
10
10

## Slide 6
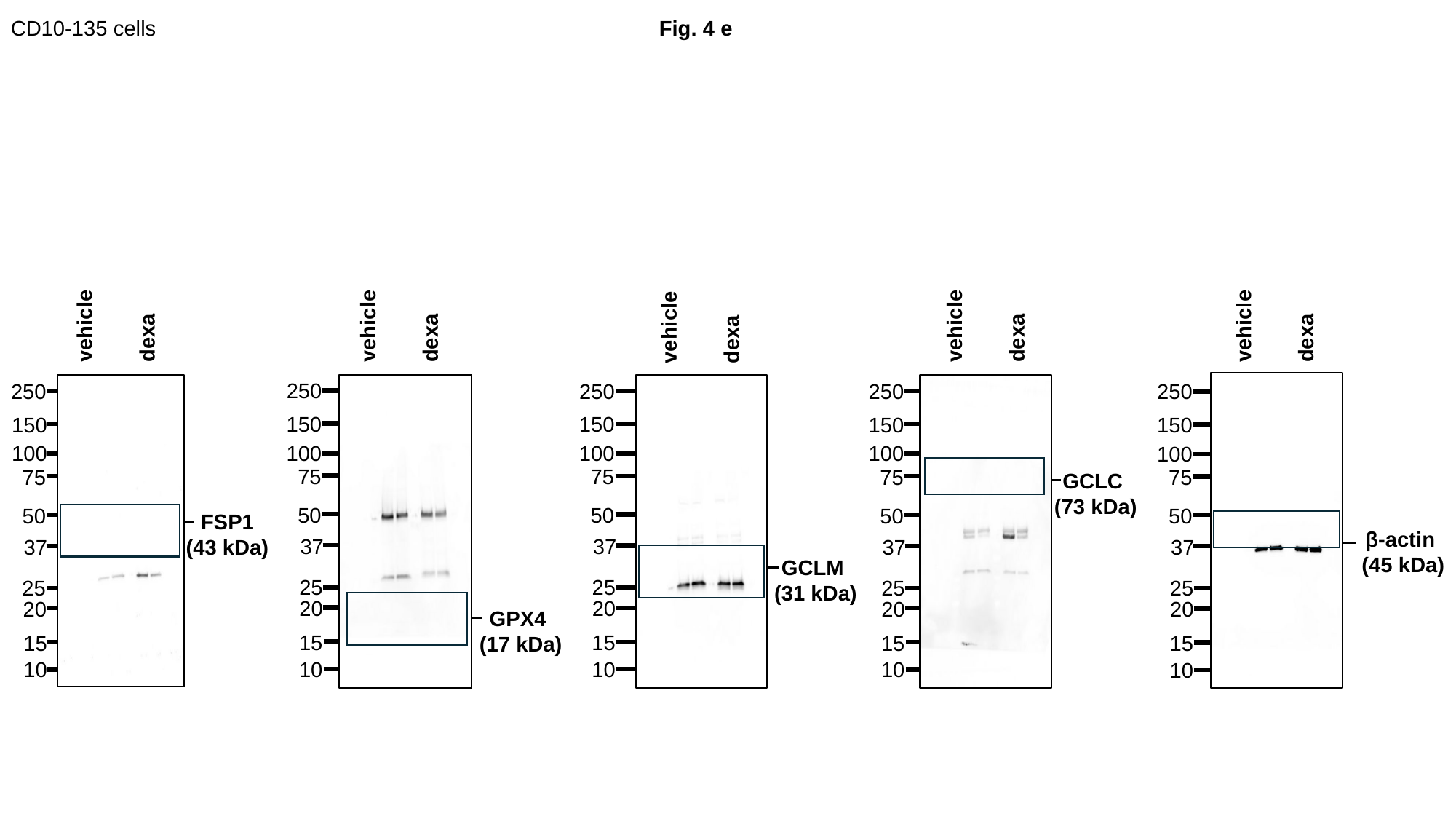

CD10-135 cells
Fig. 4 e
vehicle
vehicle
vehicle
vehicle
vehicle
dexa
dexa
dexa
dexa
dexa
250
250
250
250
250
150
150
150
150
150
100
100
100
100
100
75
75
75
75
75
GCLC
(73 kDa)
50
50
50
50
50
FSP1
(43 kDa)
β-actin
(45 kDa)
37
37
37
37
37
GCLM
(31 kDa)
25
25
25
25
25
20
20
20
20
20
GPX4
(17 kDa)
15
15
15
15
15
10
10
10
10
10

## Slide 7
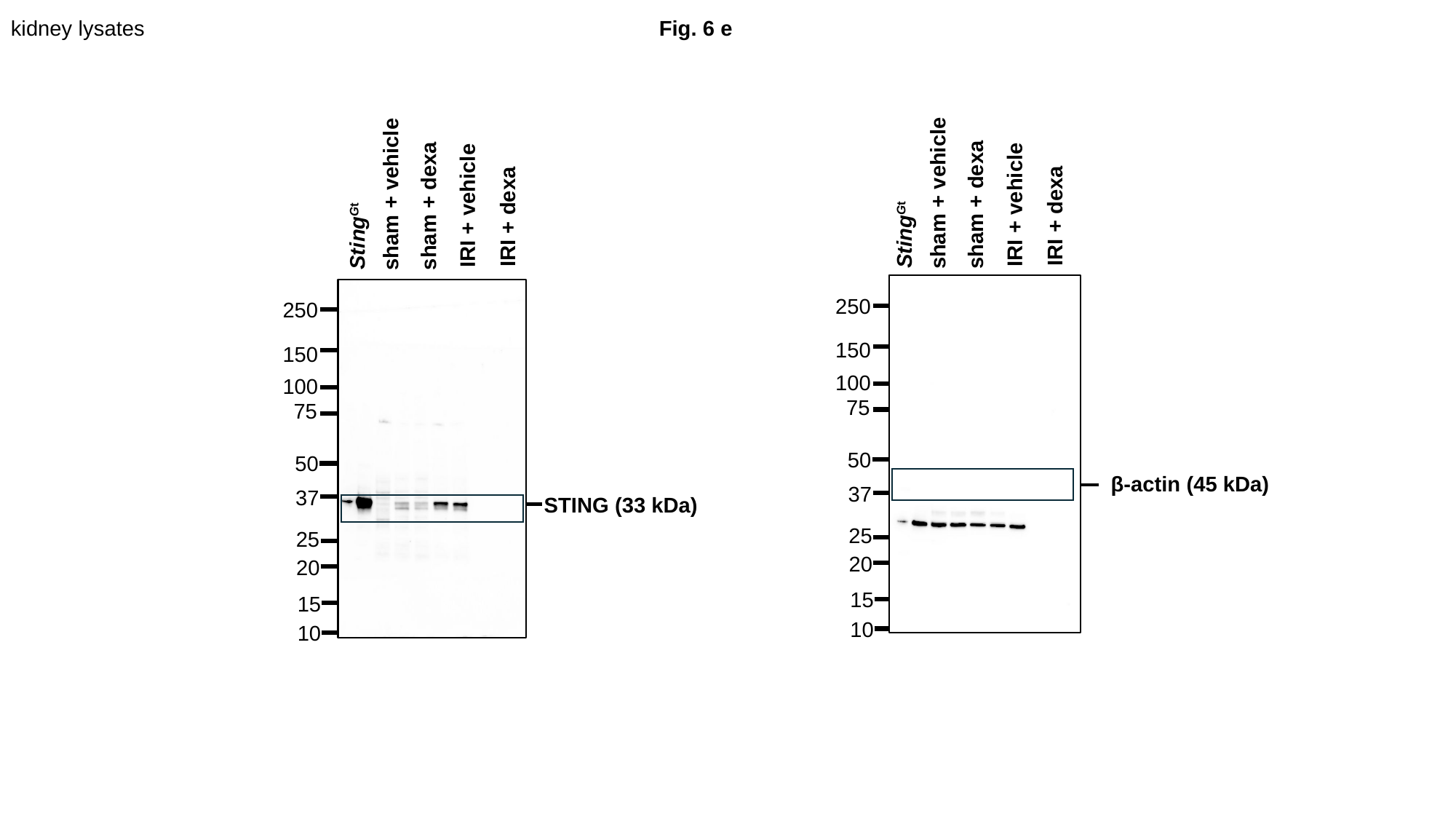

kidney lysates
Fig. 6 e
sham + dexa
sham + dexa
IRI + dexa
IRI + dexa
IRI + vehicle
IRI + vehicle
StingGt
sham + vehicle
StingGt
sham + vehicle
250
250
150
150
100
100
75
75
50
50
β-actin (45 kDa)
37
37
STING (33 kDa)
25
25
20
20
15
15
10
10

## Slide 8
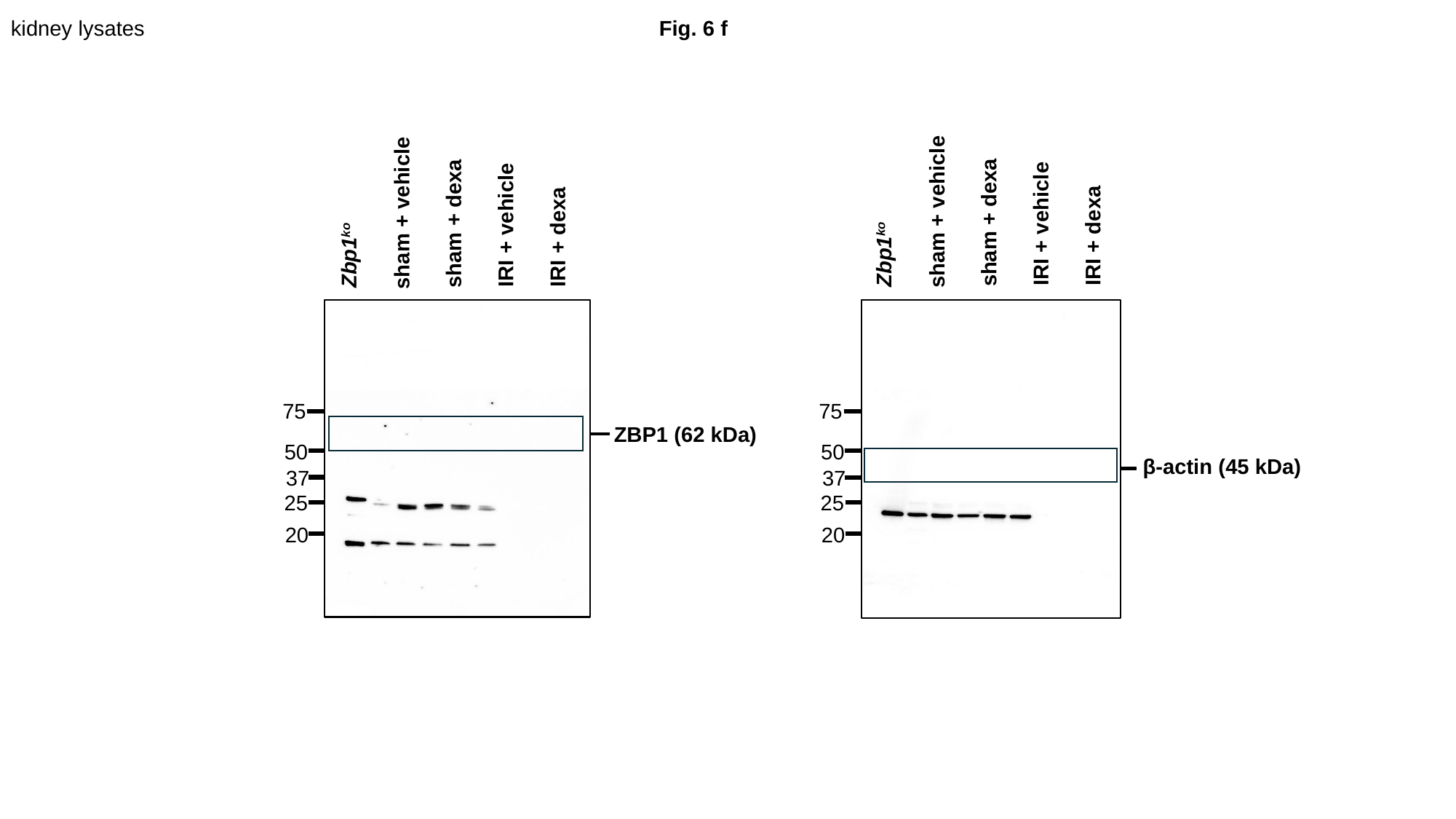

kidney lysates
Fig. 6 f
IRI + dexa
IRI + vehicle
IRI + dexa
IRI + vehicle
Zbp1ko
sham + dexa
sham + vehicle
Zbp1ko
sham + dexa
sham + vehicle
75
75
ZBP1 (62 kDa)
50
50
β-actin (45 kDa)
37
37
25
25
20
20

## Slide 9
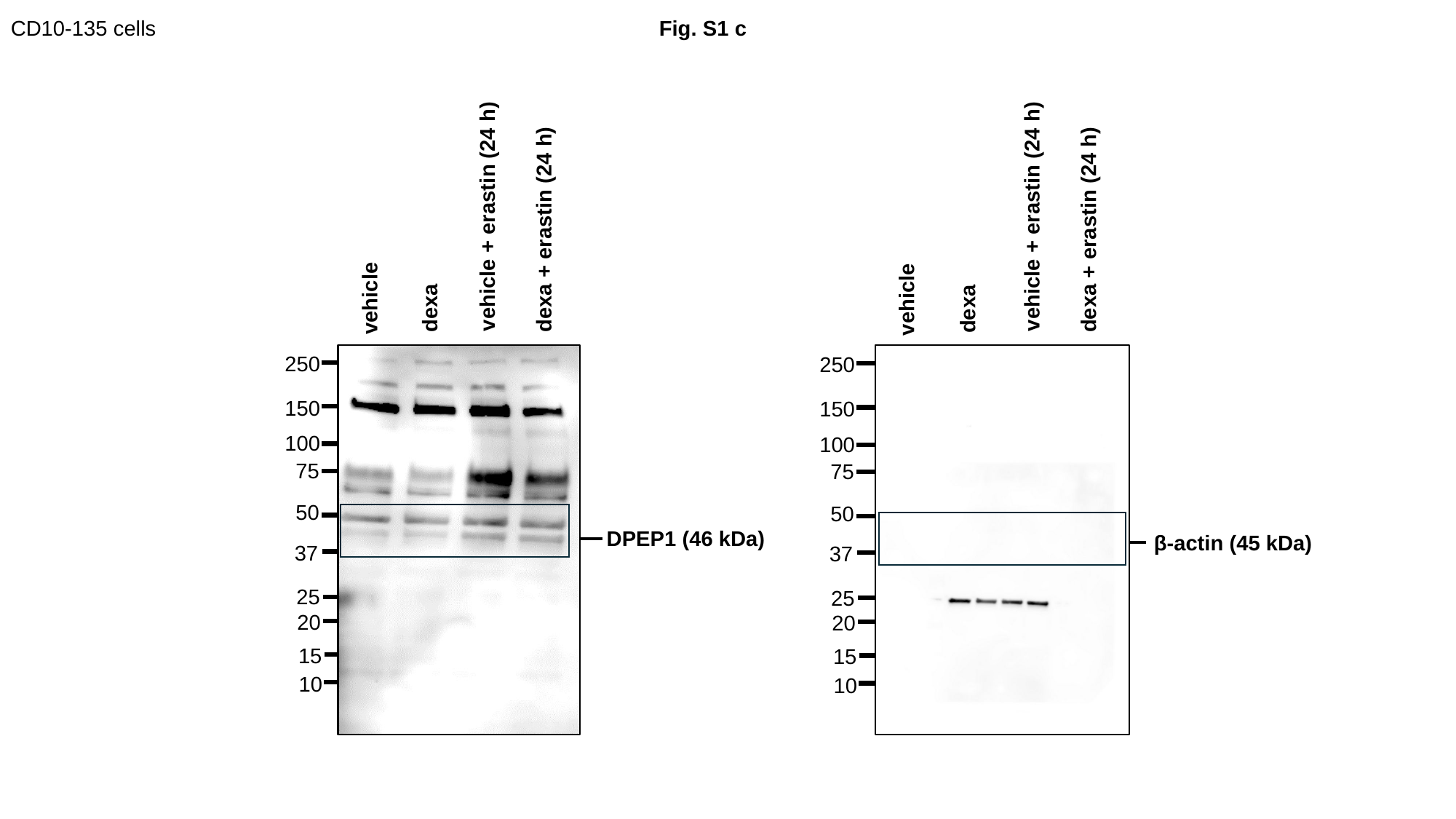

CD10-135 cells
Fig. S1 c
vehicle + erastin (24 h)
vehicle + erastin (24 h)
dexa
dexa + erastin (24 h)
dexa + erastin (24 h)
dexa
vehicle
vehicle
250
250
150
150
100
100
75
75
50
50
DPEP1 (46 kDa)
β-actin (45 kDa)
37
37
25
25
20
20
15
15
10
10

## Slide 10
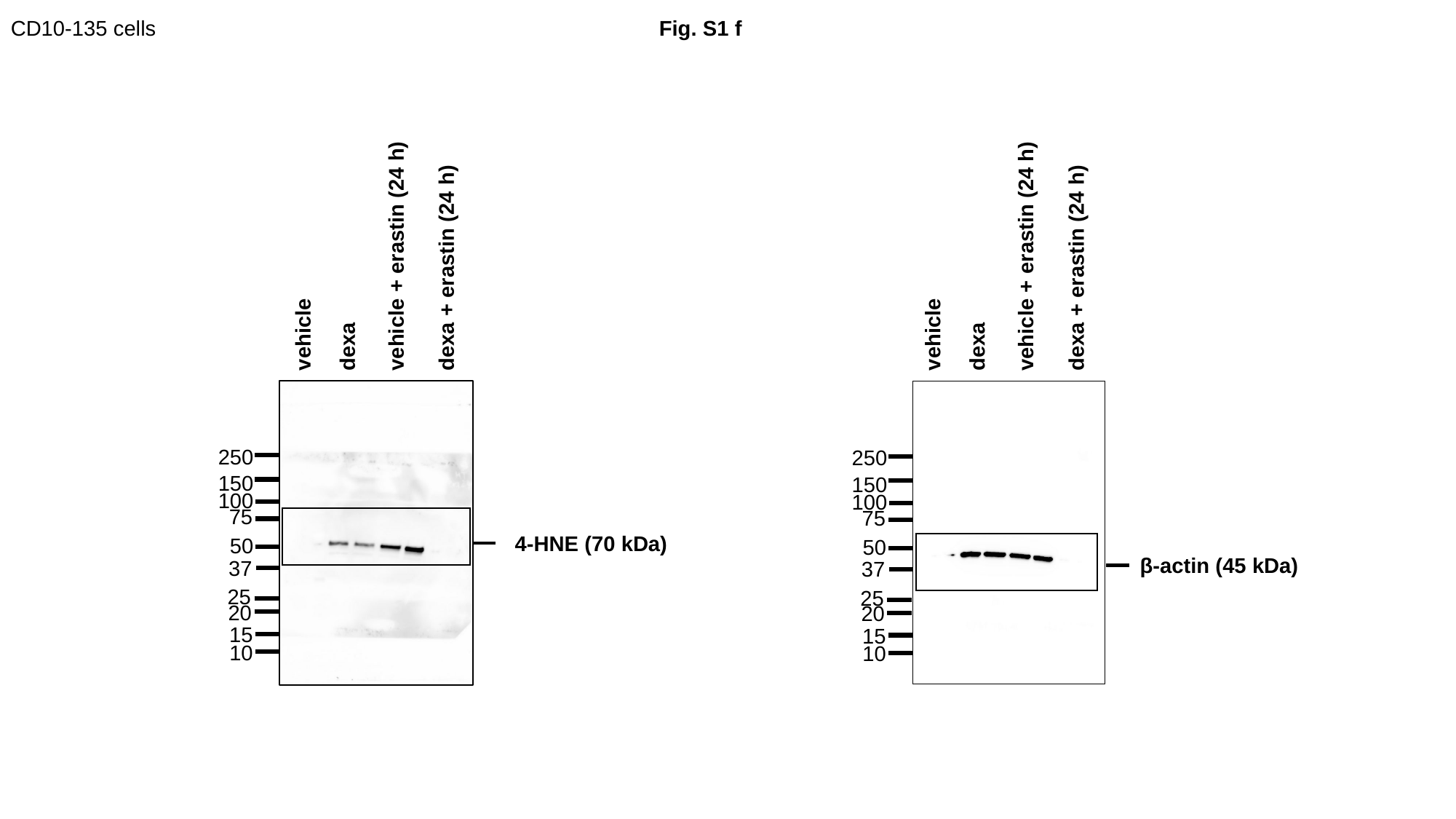

CD10-135 cells
Fig. S1 f
dexa + erastin (24 h)
dexa
vehicle + erastin (24 h)
dexa + erastin (24 h)
dexa
vehicle + erastin (24 h)
vehicle
vehicle
250
250
150
150
100
100
75
75
4-HNE (70 kDa)
50
50
β-actin (45 kDa)
37
37
25
25
20
20
15
15
10
10
